# Supplementary material for: Light-induced Kondo-like exciton-spin interaction in neodymium(II) doped hybrid perovskite
Source: Nat Commun. 2024 Jul 19;15:6084. doi: 10.1038/s41467-024-50196-1 (PMC11271502; doi:10.1038/s41467-024-50196-1)
Supplement: Supplementary file 1 — Supplementary Information [file 41467_2024_50196_MOESM1_ESM.pdf]

Supplementary Information for

Light-induced Kondo-like exciton-spin interaction in neodymium(II) doped hybrid perovskite

Xudong Xiao,[1#] Kyaw Zin Latt,[2#] Jue Gong,[1] Taewoo Kim,[3] Justin G. Connell,[3] Yuzi Liu,[2] H. Christopher Fry,[2] John E. Pearson,[2] Owen S. Wostoupal,[1] Mengyuan Li,[1] Calvin Soldan,[1] Zhenzhen Yang,[4] Richard D. Schaller,[2] Benjamin T. Diroll,[2*] Saw Wai Hla,[2*] Tao Xu[1*]

[1] Department of Chemistry and Biochemistry, Northern Illinois University, DeKalb, Illinois 60115, United States

[2] Center for Nanoscale Materials, Argonne National Laboratory, Lemont, Illinois 60439, United States

[3] Materials Science Division, Argonne National Laboratory, Lemont, Illinois 60439, United States

[4] Chemical Sciences and Engineering Division, Argonne National Laboratory, Lemont, Illinois 60439, United States

# These authors contributed equally to this work.

*Corresponding author: Benjamin T. Diroll, bdiroll@anl.gov; Saw Wai Hla, shla@anl.gov; Tao Xu, txu@niu.edu

This PDF file includes:

Supplementary Figs. 1 to 28

Supplementary Tables 1 to 3

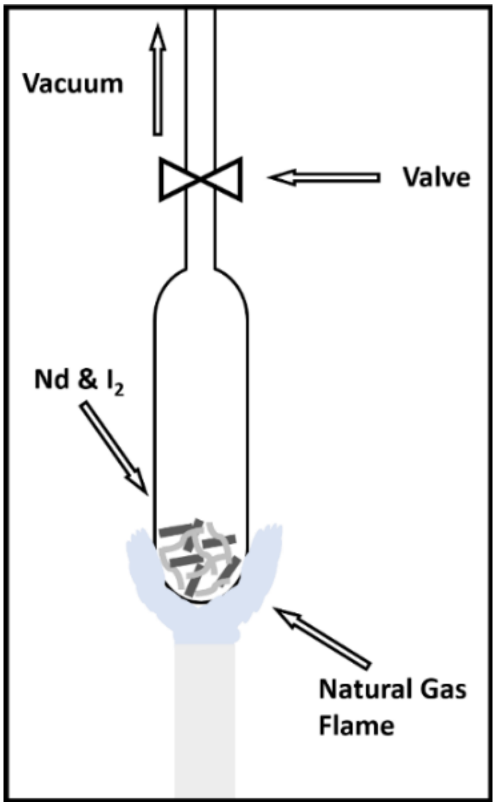

Supplementary Fig. 1. Schematic diagram of NdI_2 synthesis process.

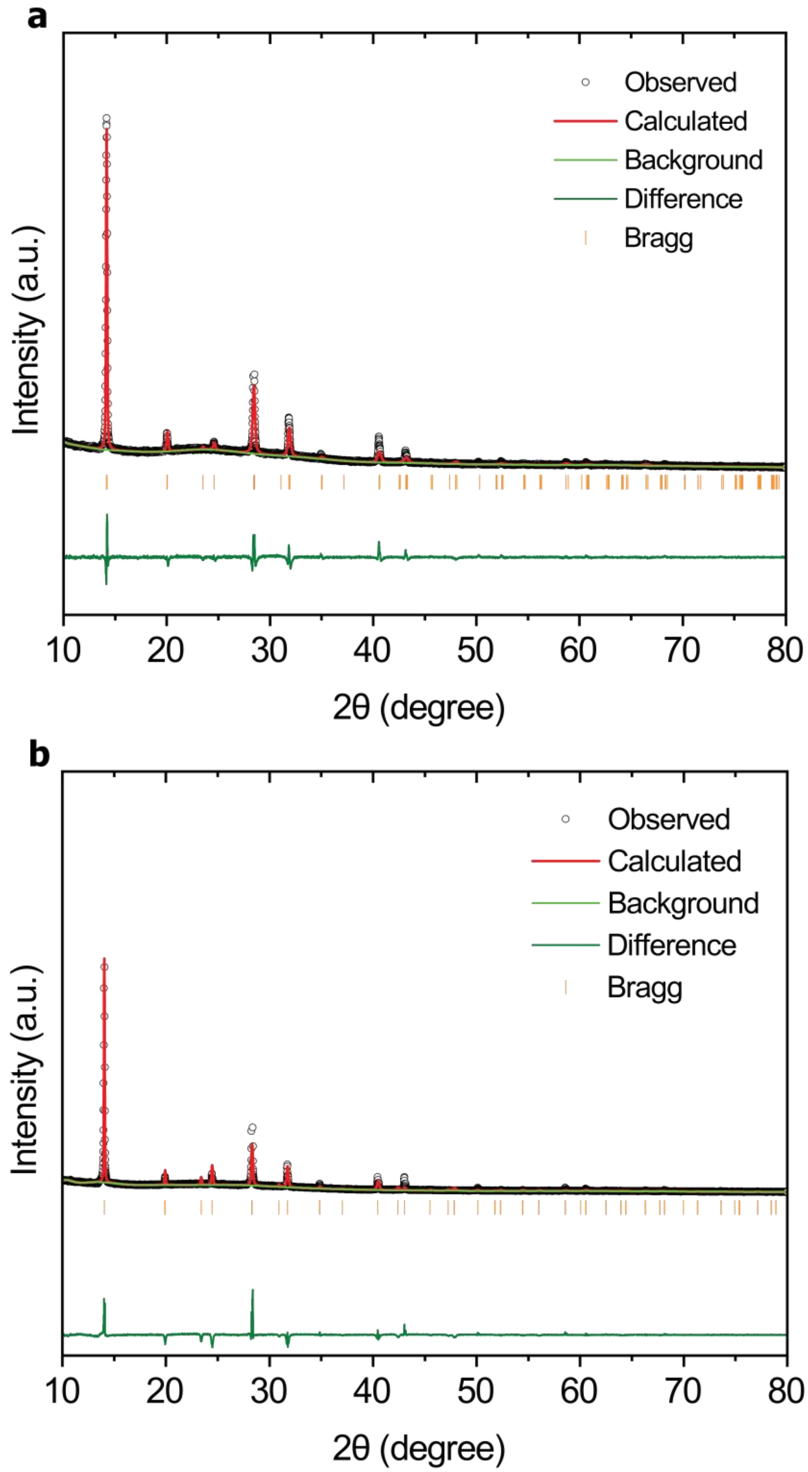

Supplementary Fig. 2. XRD refinement. a, pristine $MAPbI_3$ film. **b**, 2%Nd:$MAPbI_3$ film.

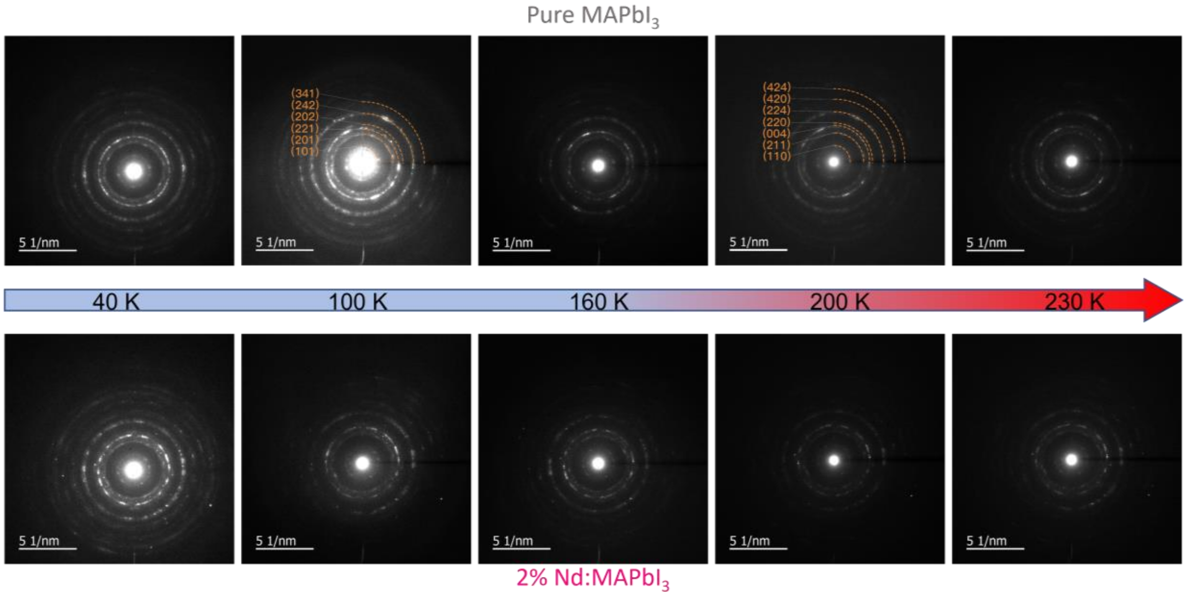

Supplementary Fig. 3. SAED patterns of pristine $MAPbI_3$ film (top row) along the heating profile from 40 K to 230 K; and the corresponding SAED patterns of 2%Nd:$MAPbI_3$ film (bottom row) along the same heating profile from 40K to 230 K.

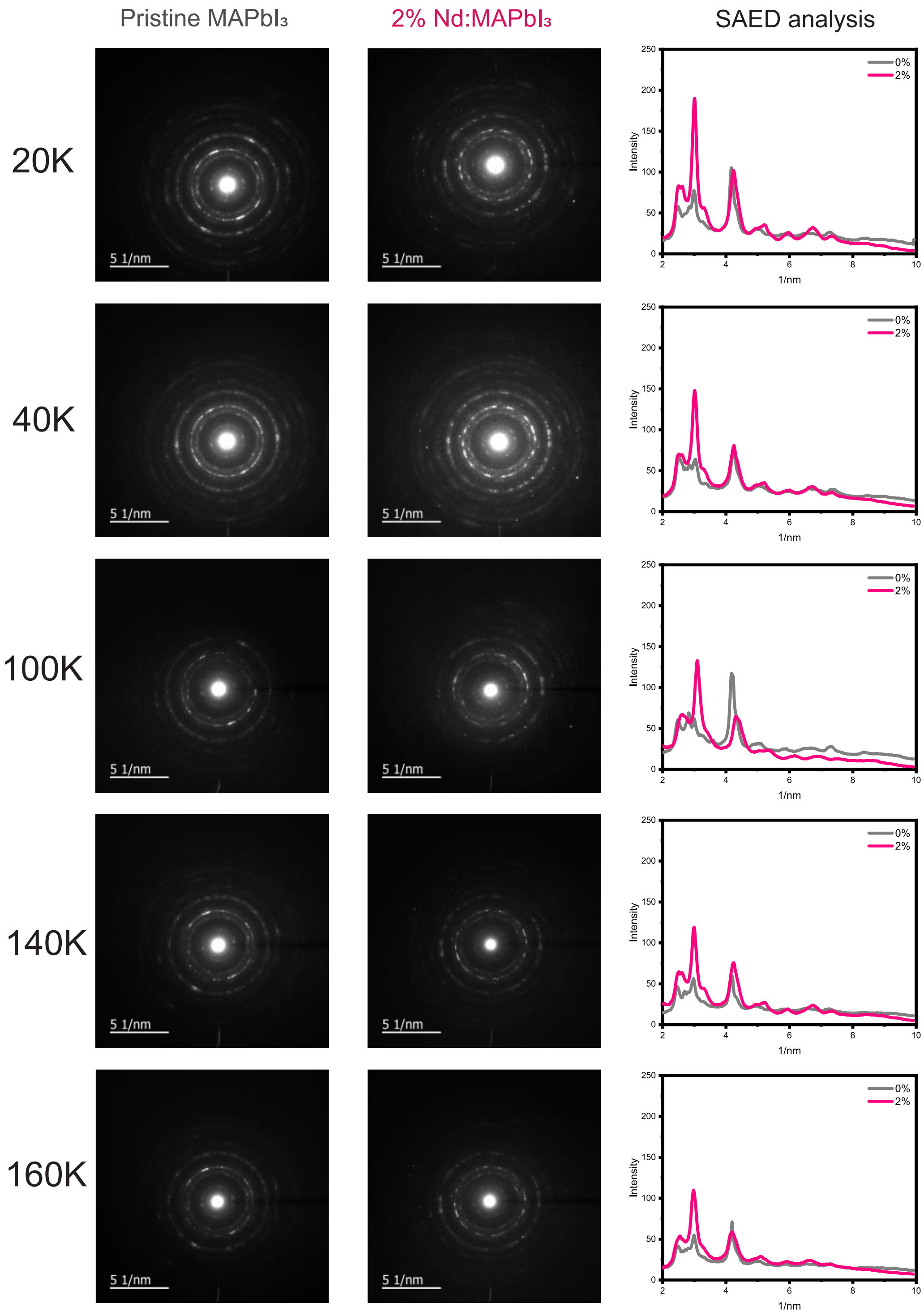
Pristine MAPbI3
2% Nd:MAPbI3
SAED analysis
20K
40K
100K
140K
160K
5 1/nm
Intensity
1/nm
0%
2%

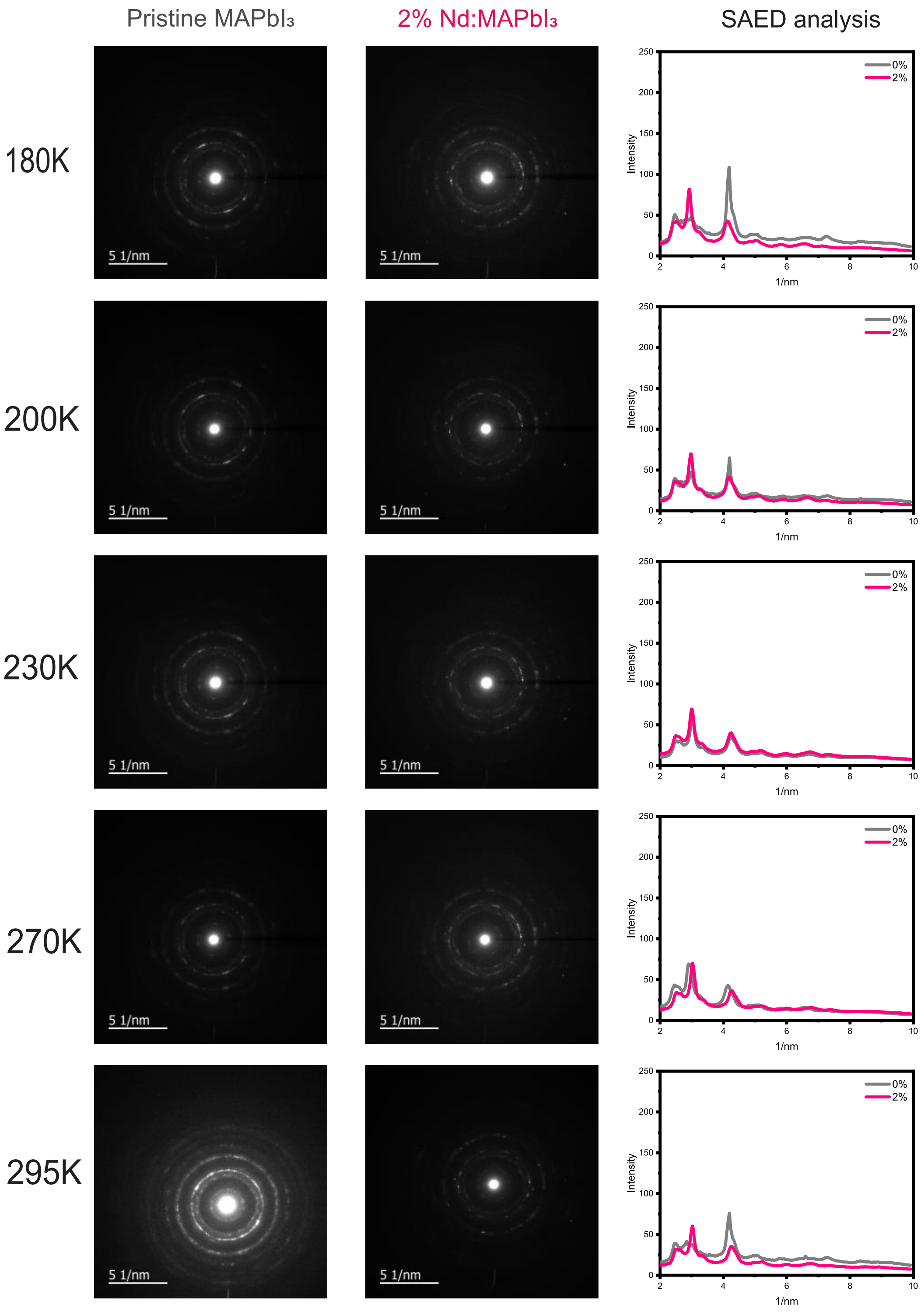

Supplementary Fig. 4. Comparison of the SAED patterns between pristine $MAPbI_3$ and 2%Nd:$MAPbI_3$ at different temperatures from 20 K to room temperature, and corresponding SAED analysis by using Digital Micrograph software. Based on the SAED patterns analyzed in Digital Micrograph software, the crystal structure of pristine $MAPbI_3$ and 2% Nd:$MAPbI_3$ remain

similar at the same temperature. The characteristic peak of the orthorhombic phase at (221) crystal plane was found at 100 K. However, at 200 K, this crystal planes disappear while other planes characteristic to the tetragonal phase are present including (004) and (220) planes. We thus conclude that the phase transition from orthorhombic to tetragonal is around 100K to 160 K, in agreement with reports in literature.

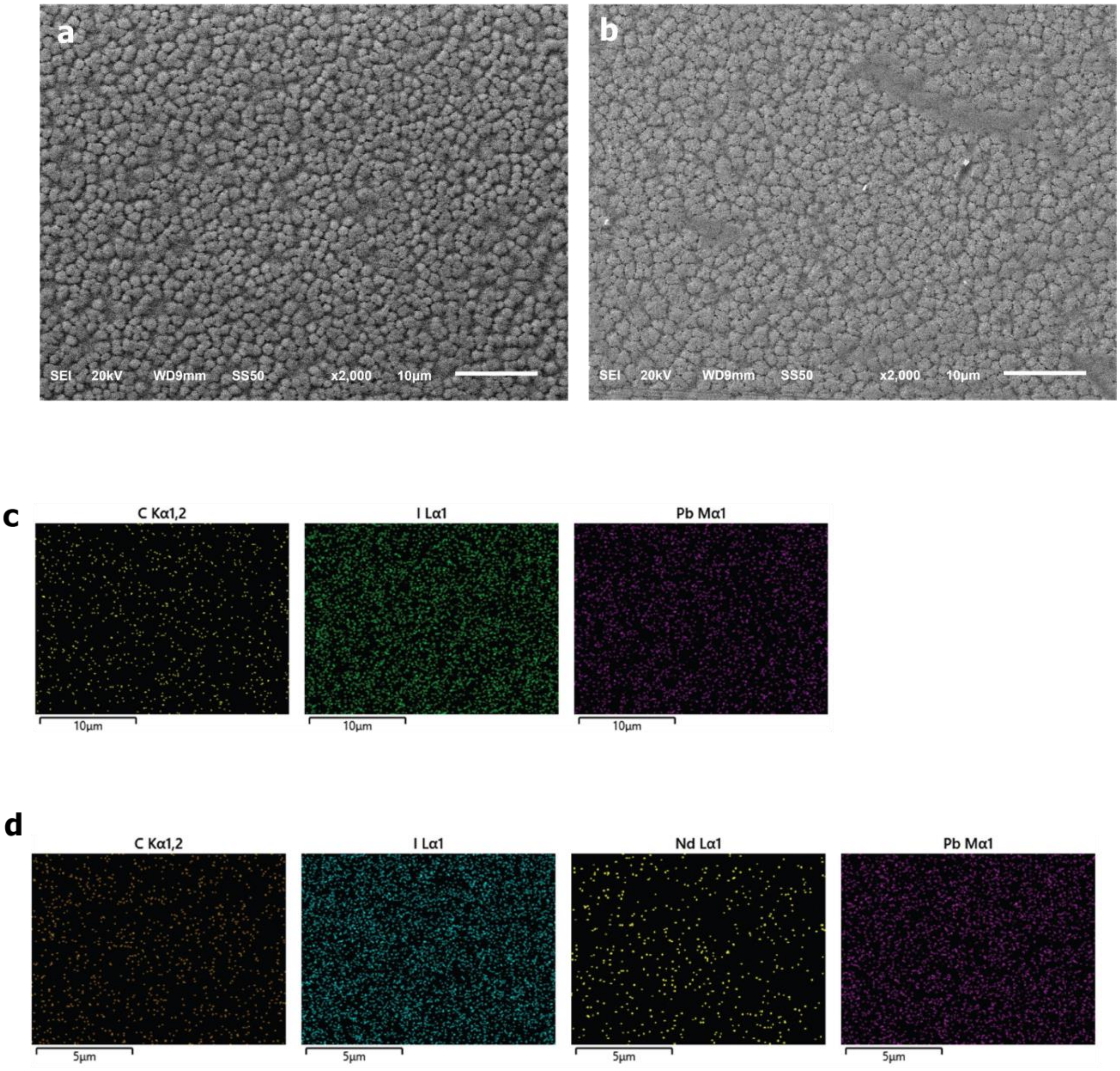

Supplementary Fig. 5. SEM film morphology study. a, pristine $MAPbI_3$ film and **b**, 2%Nd:$MAPbI_3$; **EDX elemental mapping of C, I, Nd, Pb. c**, pristine $MAPbI_3$ film and **d**, 2%Nd:$MAPbI_3$.

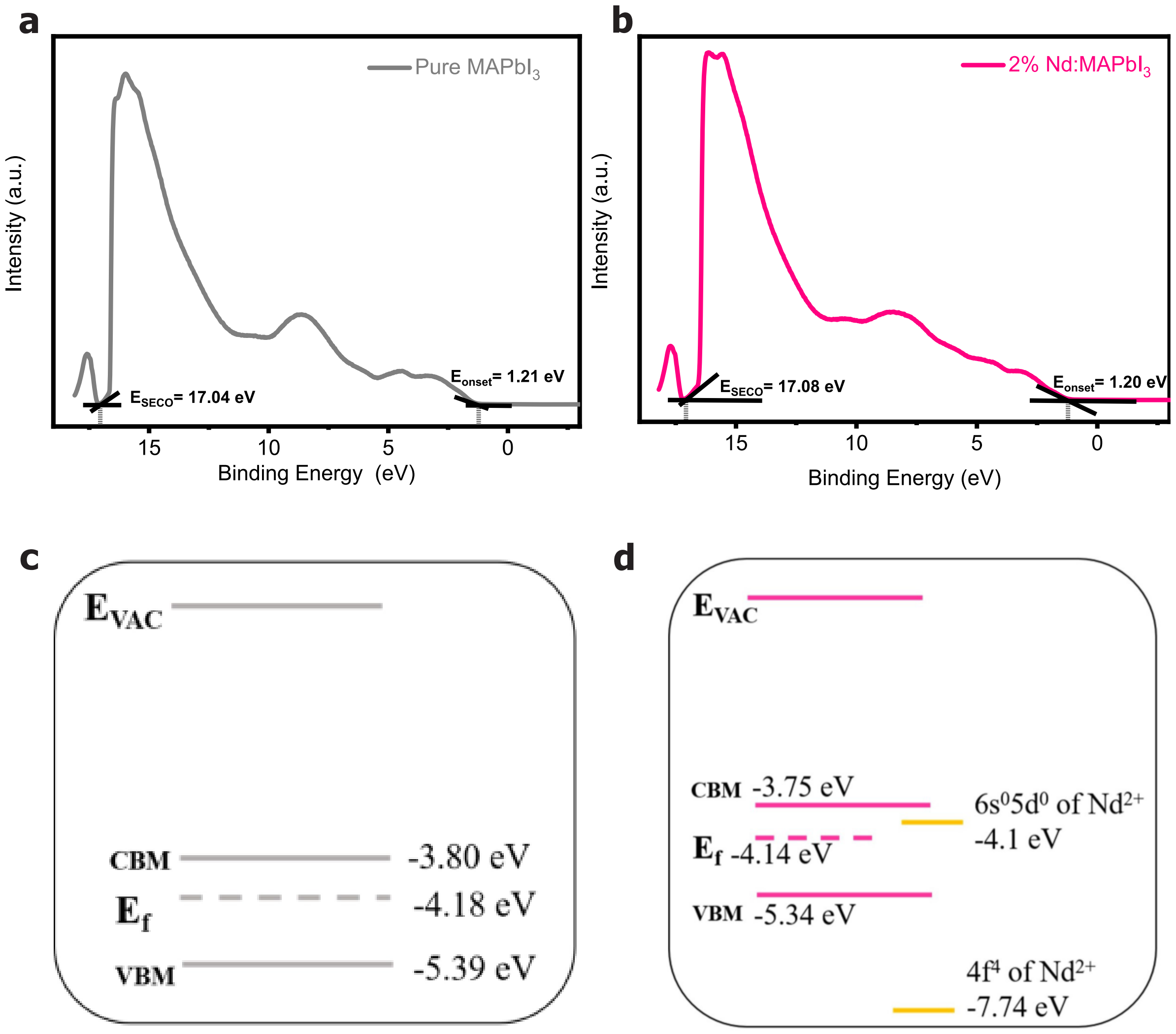

Supplementary Fig. 6. The determination of VBM and Fermi energy (E_f) of the pristine $MAPbI_3$ and 2%Nd:$MAPbI_3$ from their respective UPS spectra. Fermi levels (E_f) can be calculated from the formula $E_f = 21.22$ eV − E_{SECO}, where E_{SECO} is the secondary electron cut-off energy, obtained as the value of the intersection of the two linear fitting curves. The valence band maximum (VBM) can be calculated from the formula $E_{VBM} = 21.22$ eV −(E_{SECO} − E_{onset}), where E_{onset} is the onset electron energy, obtained as the intersection of the two linear fitting curves.

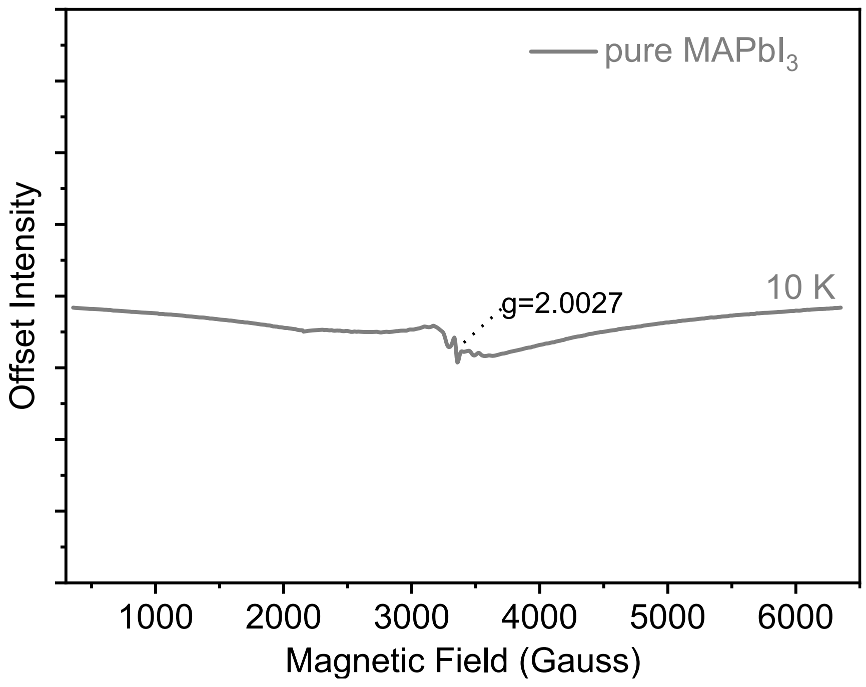

Supplementary Fig. 7. CW-EPR of ground powder of pristine $MAPbI_3$ at 10 K. Showing no signal at the low field but the high field signal at g=2.0027 still exists.

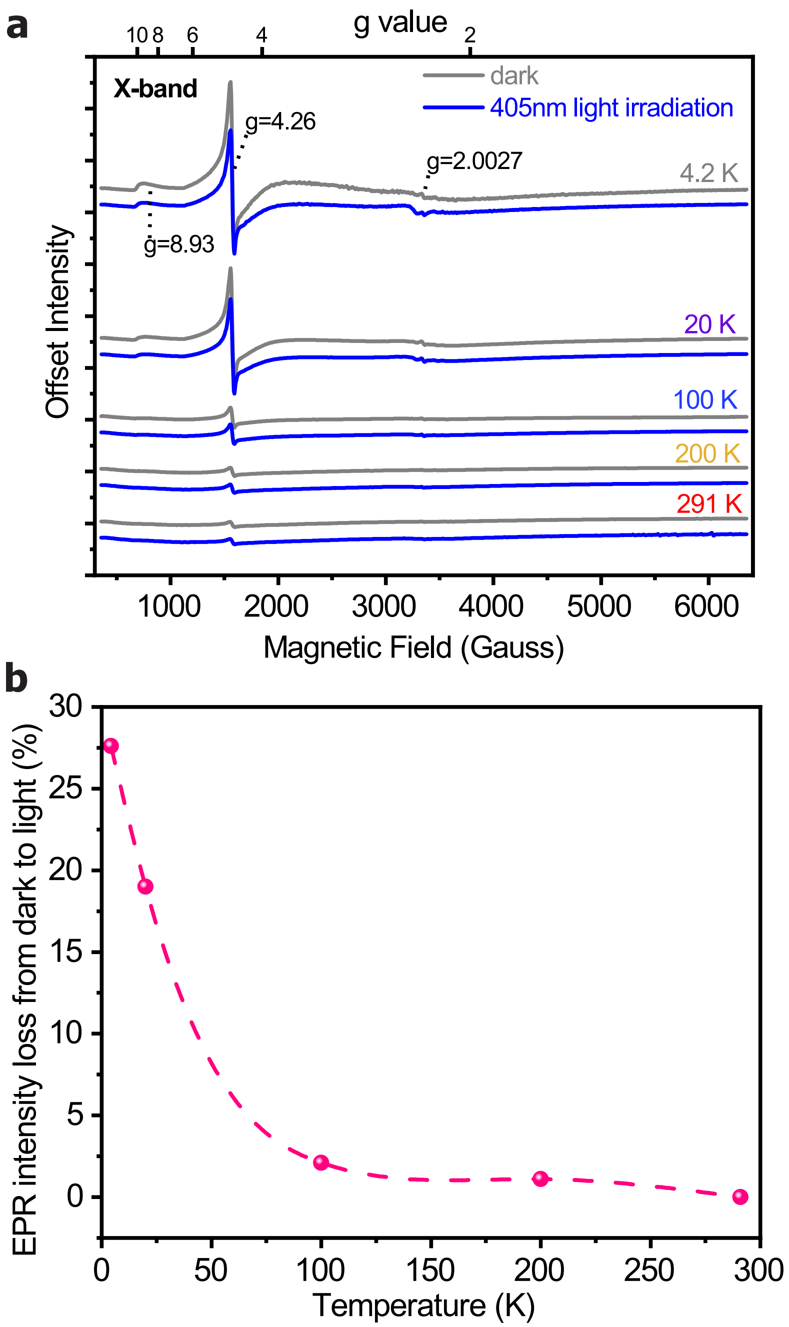

Supplementary Fig. 8. Temperature-dependent CW-EPR in dark and light conditions. a, Temperature-dependent CW-EPR of 2%Nd:$MAPbI_3$ in dark and under 405nm laser radiation (100mW). **b**, Relative percentile EPR signal loss from dark to light conditions at different temperatures. The line is to guide the eye.

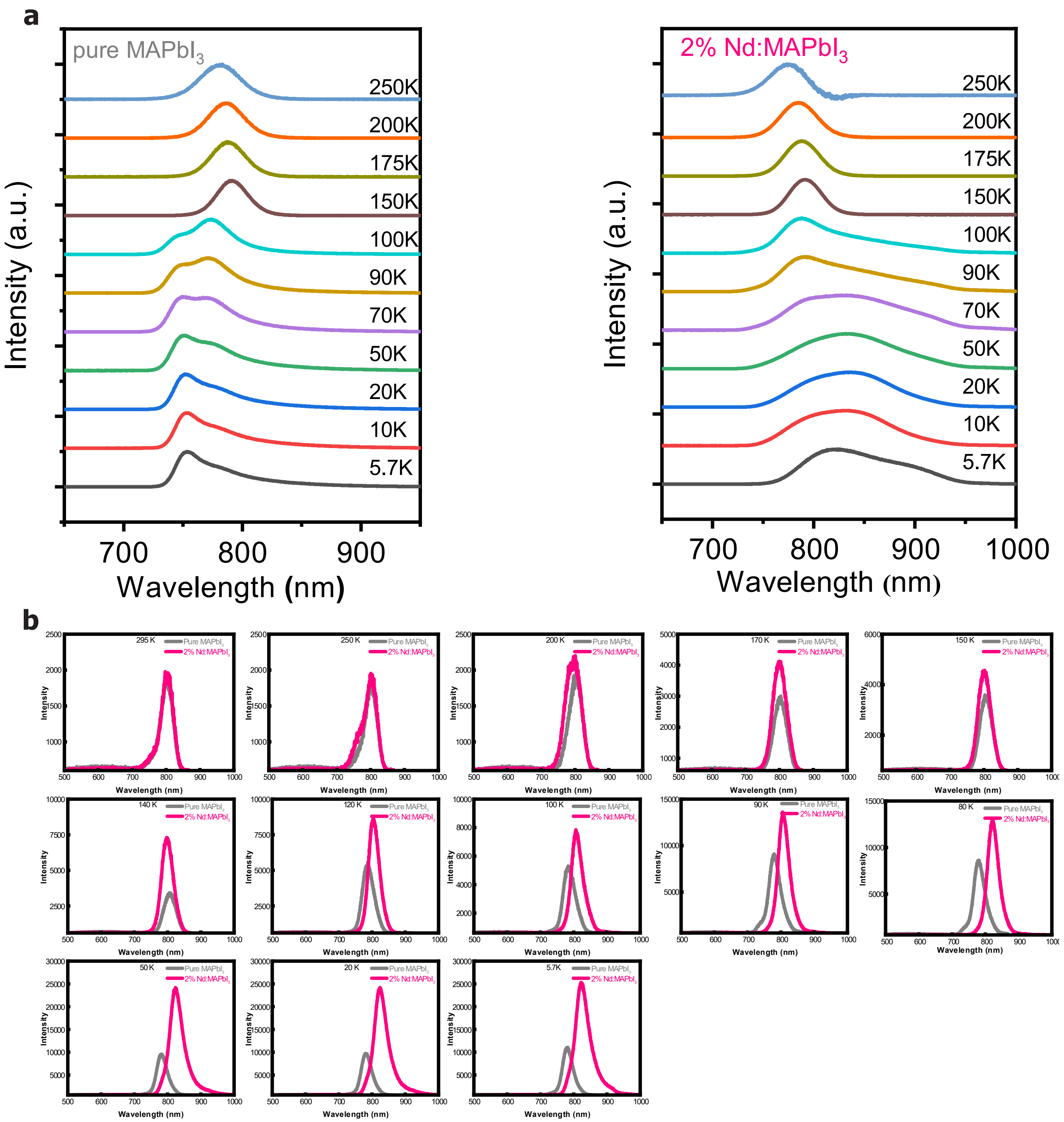

Supplementary Fig. 9. Temperature-dependent steady-state PL position and intensity analysis. a, Temperature-dependent steady-state PL spectra (normalized) for pristine $MAPbI_3$ film (left) and 2%Nd:$MAPbI_3$ film (right). **b**, side-by-side comparison of steady-state PL spectra at different temperatures for pristine $MAPbI_3$ film and 2%Nd:$MAPbI_3$ film, all excited at the same condition of fluence=0.88 μJ/cm^2 (not normalized).

pristine $MAPbI_3$, 0.88uJ/cm²

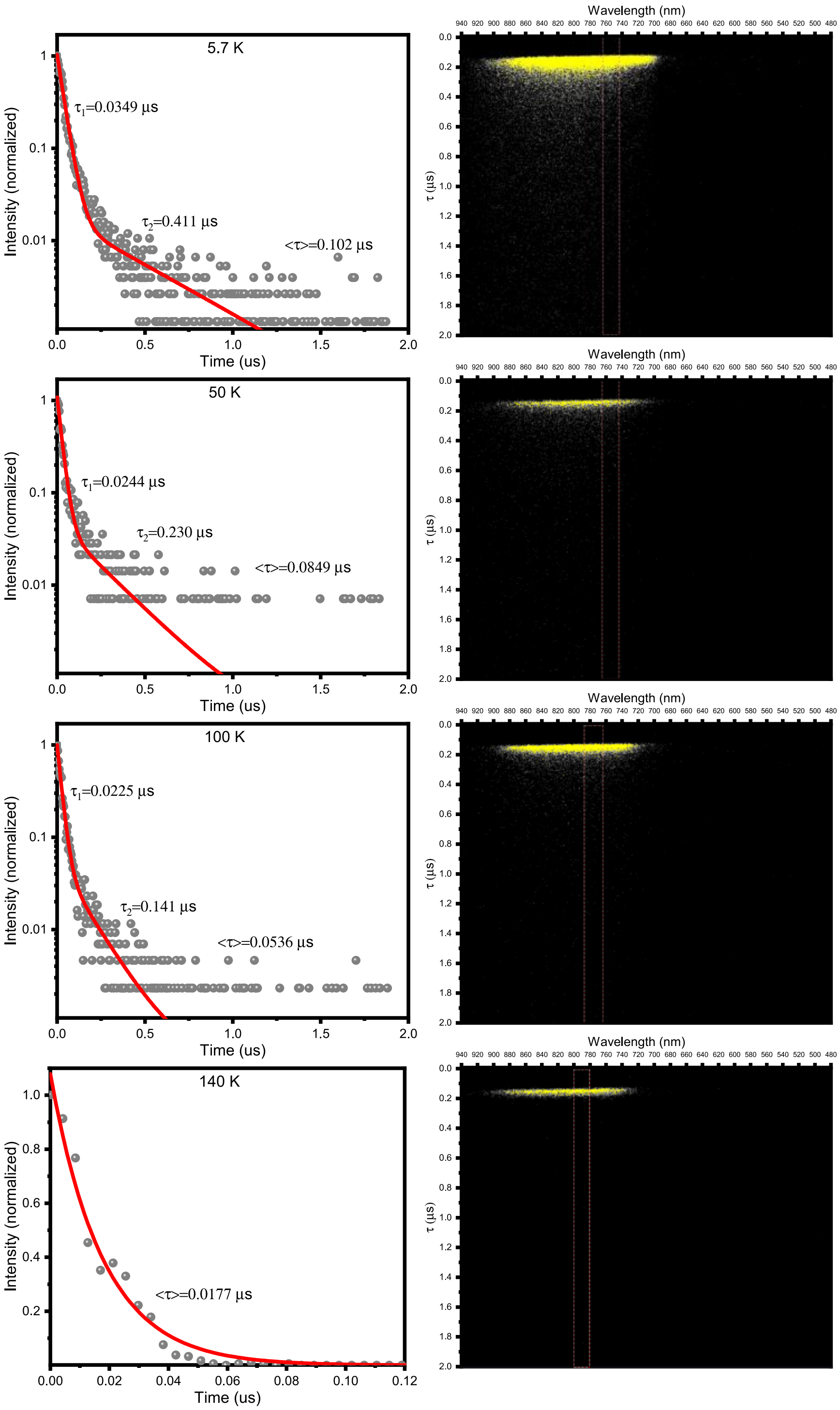

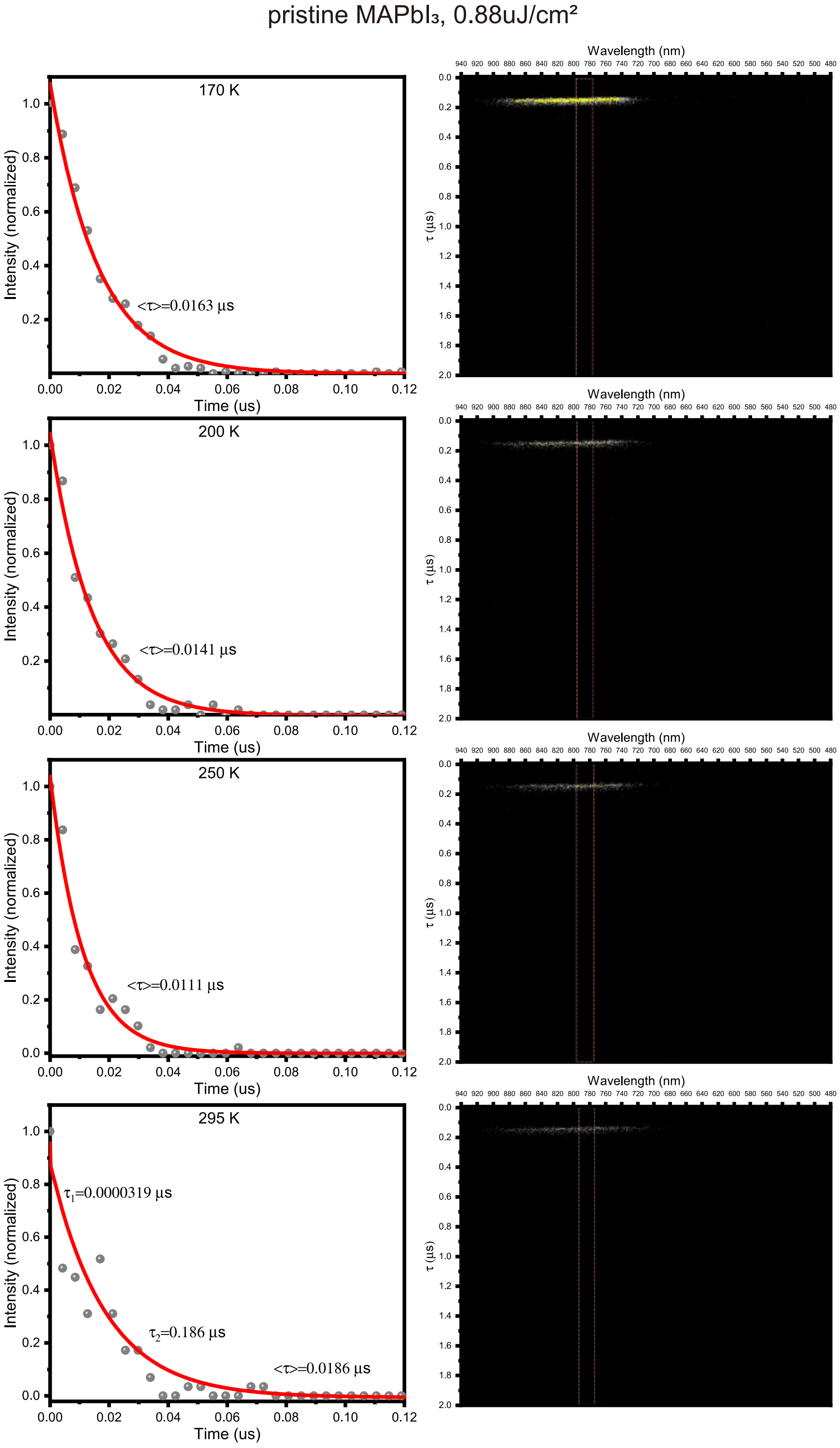

Supplementary Fig. 10. Original time-resolved PL decays of pristine $MAPbI_3$ (i.e. 0% Nd(II) doping) acquired at 5.7K - 295 K by streak camera and the fitting details. The fluence — 0.88 μJ/cm^2 is equivalent to 1760 μW/cm^2.

20ppm-Nd:$MAPbI_3$, 0.88uJ/cm²

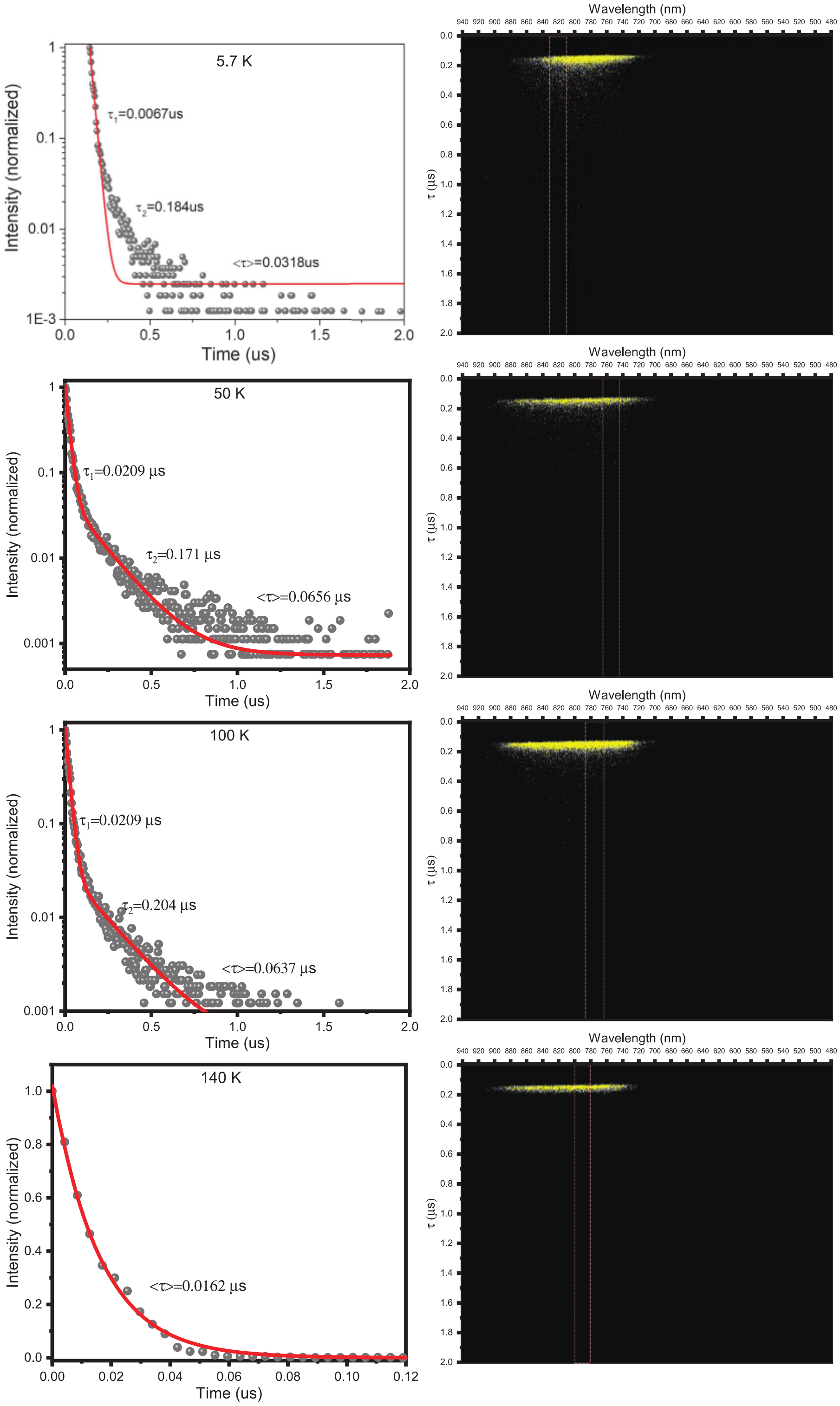

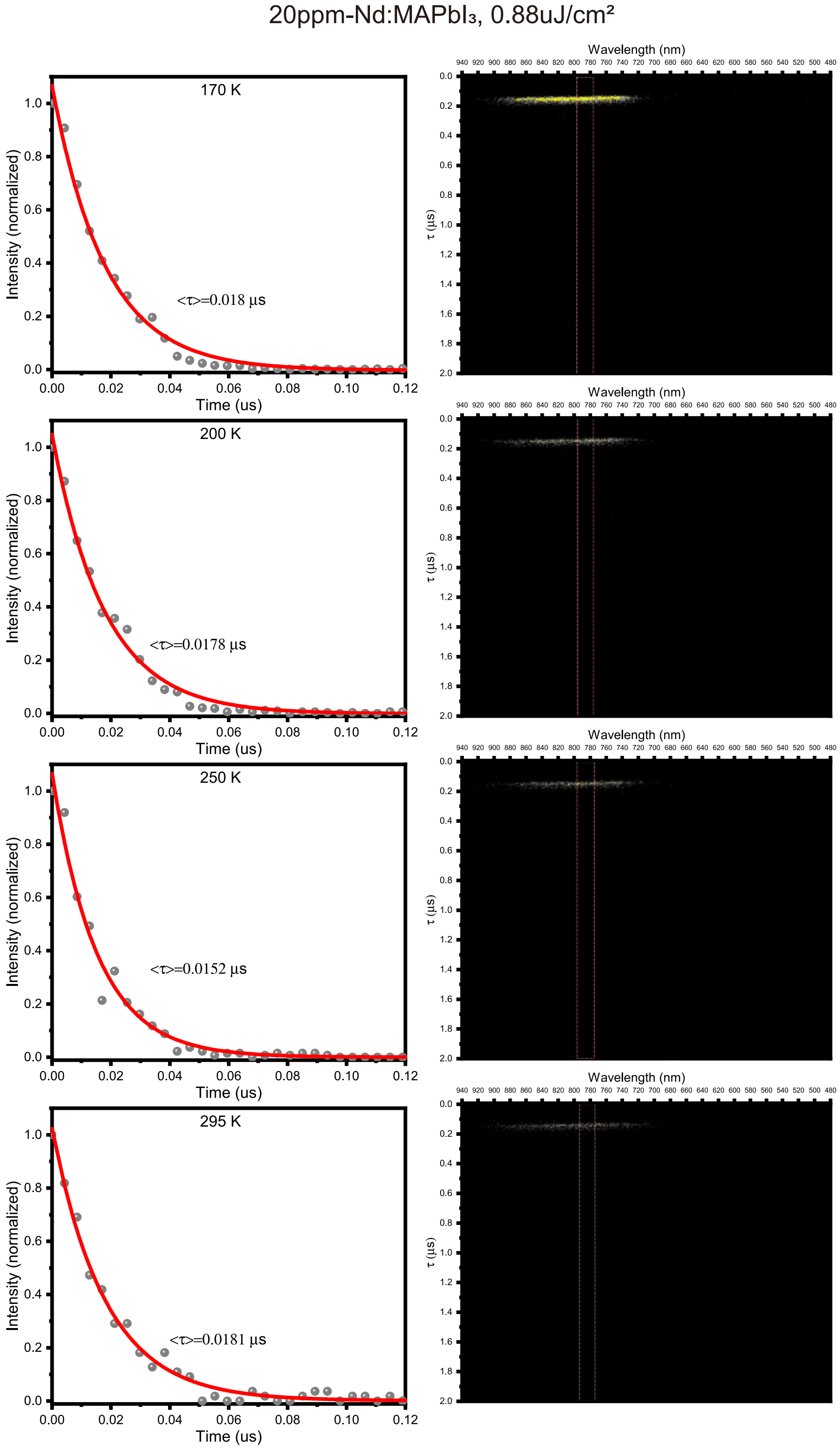

Supplementary Fig. 11. Original time-resolved PL decays of 20ppm-Nd:$MAPbI_3$ (i.e. 20 ppm Nd(II) doping) acquired at 5.7 K – 295 K by streak camera and the fitting details. The fluence — 0.88 μJ/cm^2 is equivalent to 1760 μW/cm^2.

20ppm-Nd:$MAPbI_3$, 0.11uJ/cm²

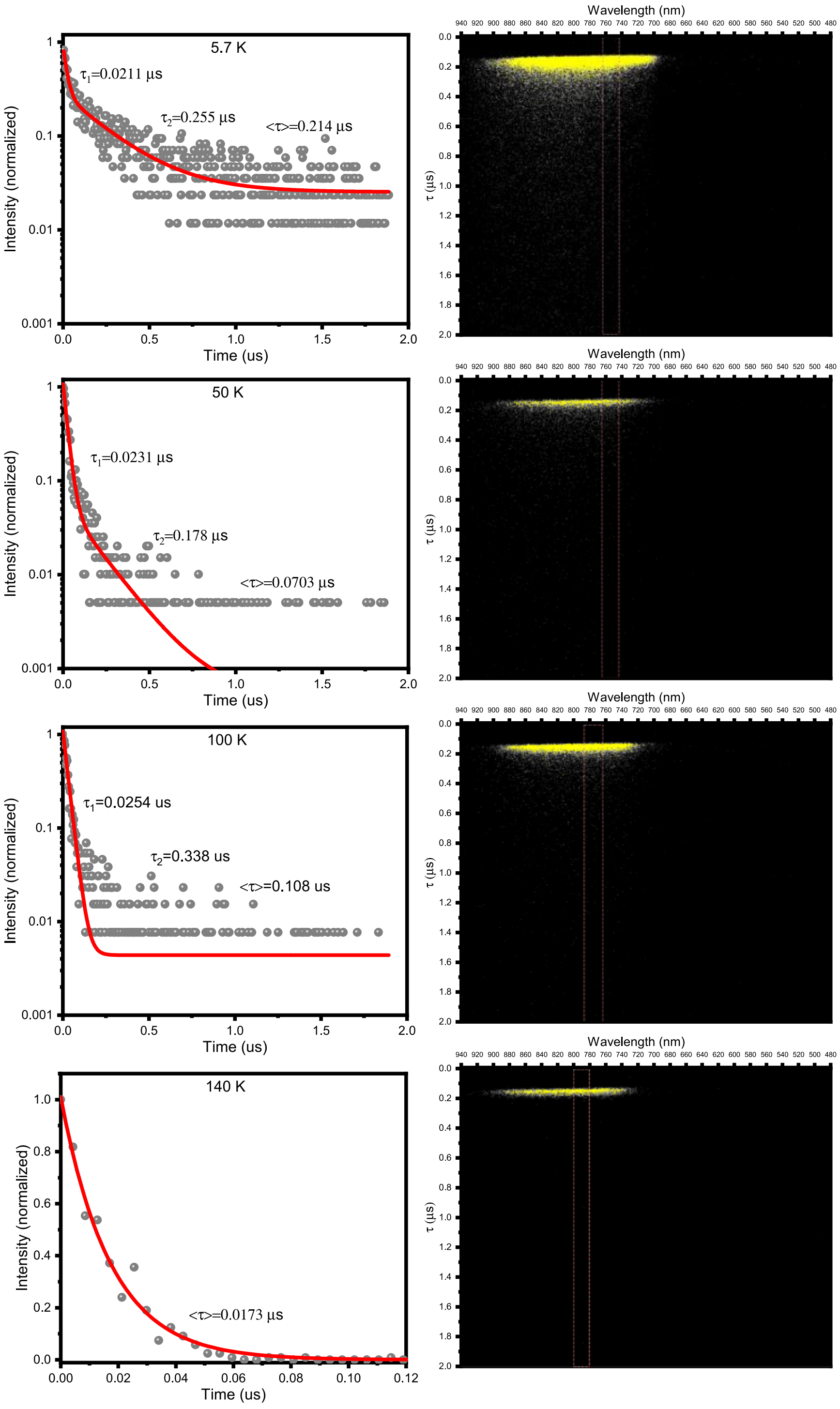

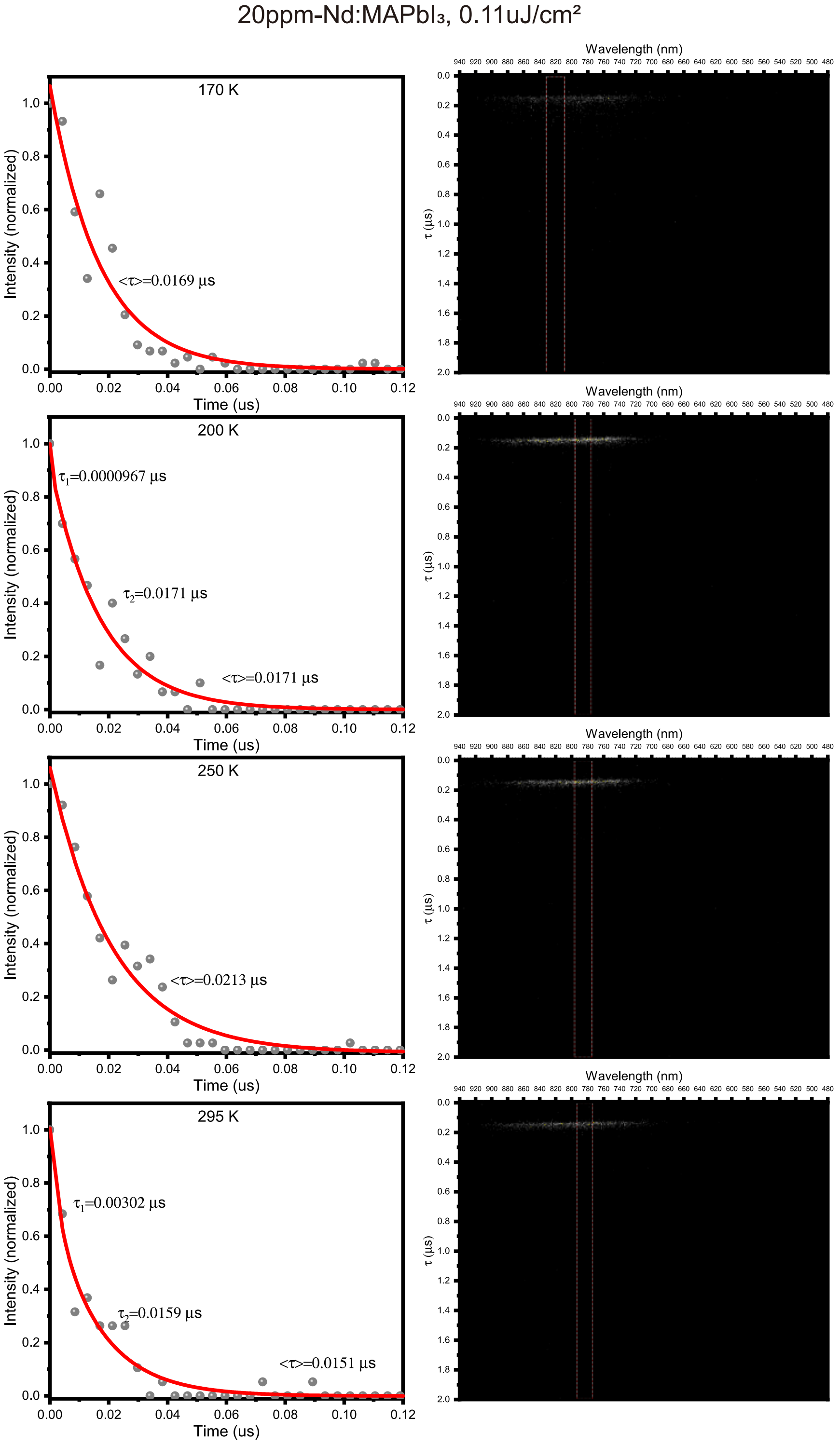

Supplementary Fig. 12. Original time-resolved PL decays of 20ppm-Nd:$MAPbI_3$ (i.e. 20 ppm Nd(II) doping) acquired at 5.7 K – 295 K by streak camera and the fitting details. The fluence — 0.11 μJ/cm^2 is equivalent to 220 μW/cm^2.

2%Nd:MAPbI₃, 0.88uJ/cm²

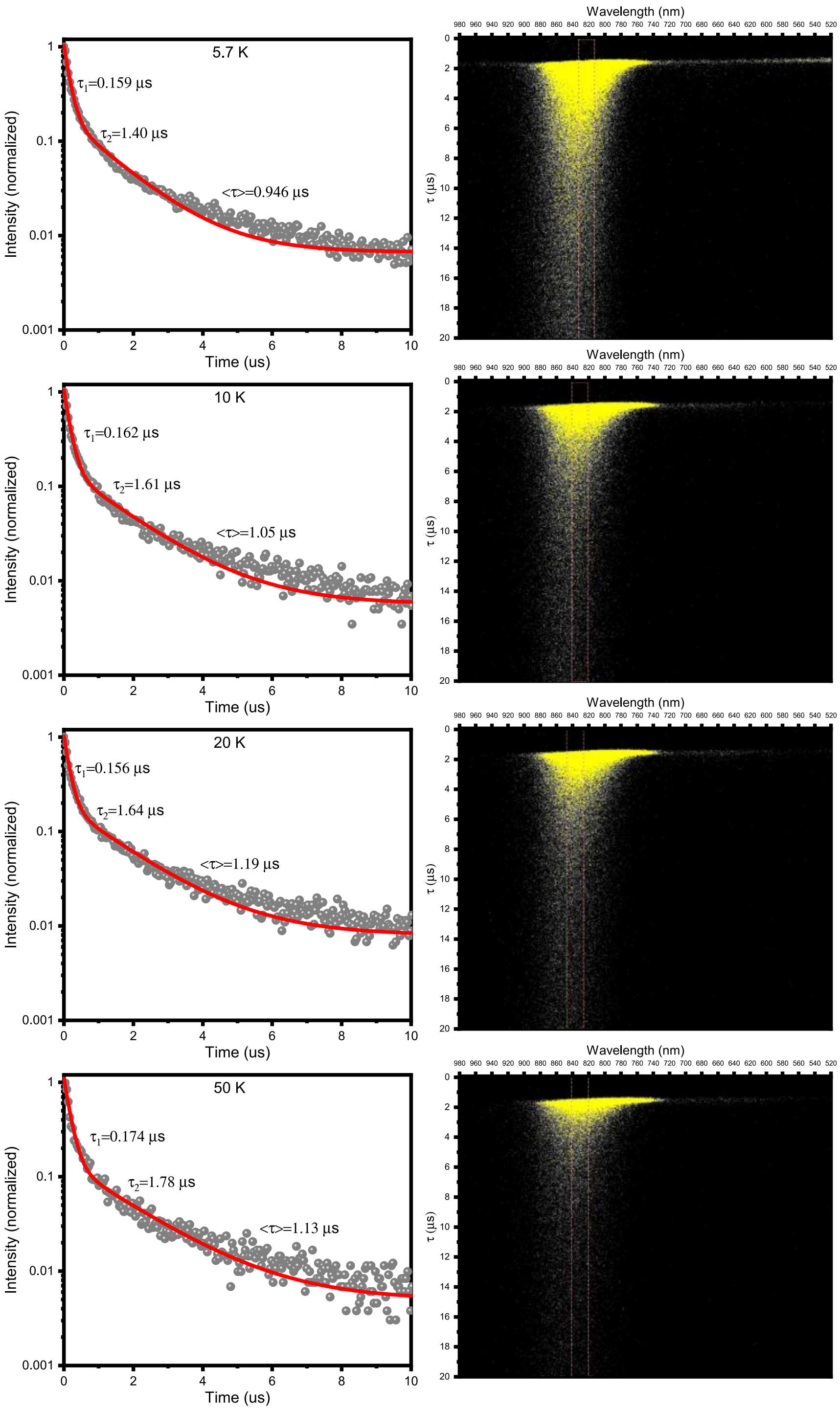

2%Nd:$MAPbI_3$, 0.88uJ/cm²

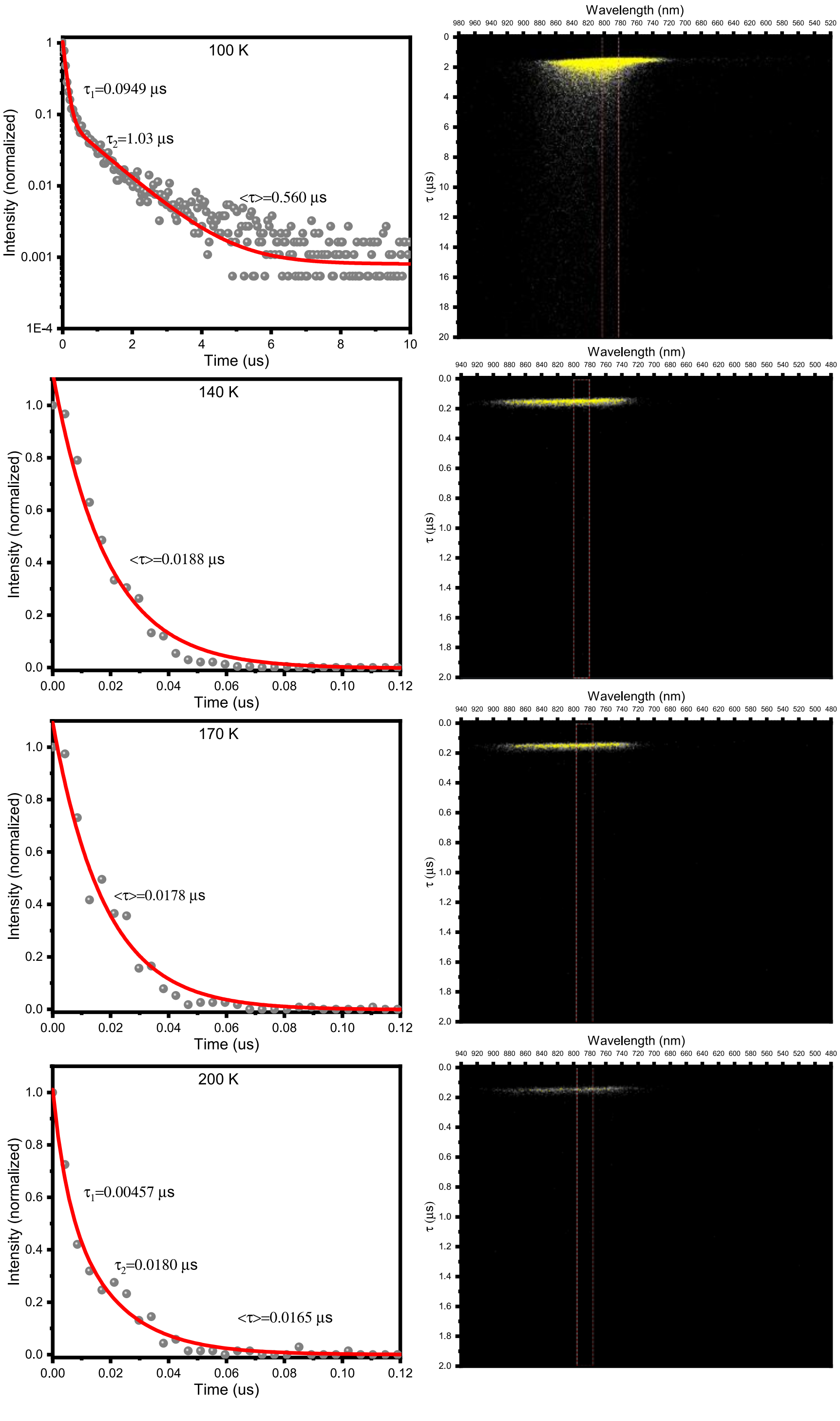

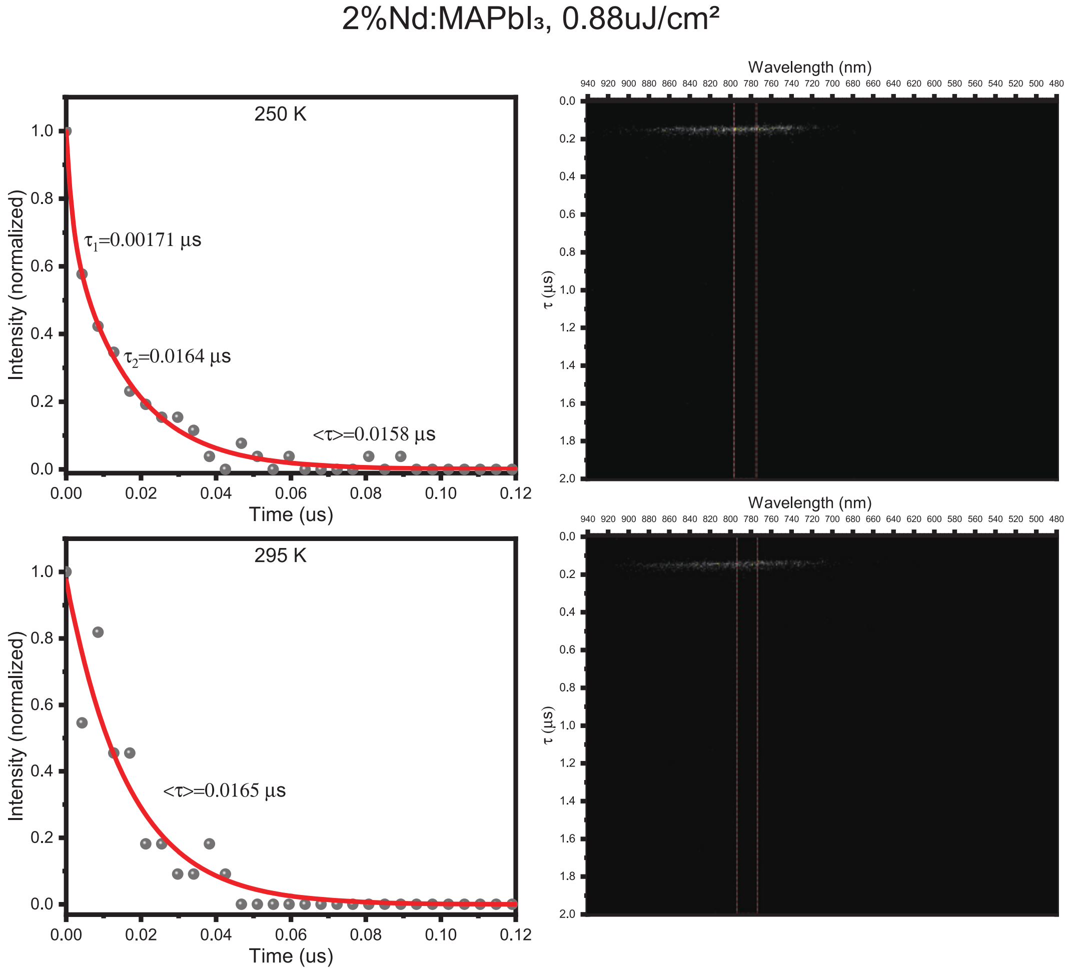

Supplementary Fig. 13. Original time-resolved PL decays of 2%Nd:$MAPbI_3$ (i.e. 2% Nd(II) doping) acquired at 5.7 K - 295 K by streak camera and the fitting details. The fluence — 0.88 μJ/cm^2 is equivalent to 1760 μW/cm^2.

2%Nd:$MAPbI_3$, 0.11uJ/cm²

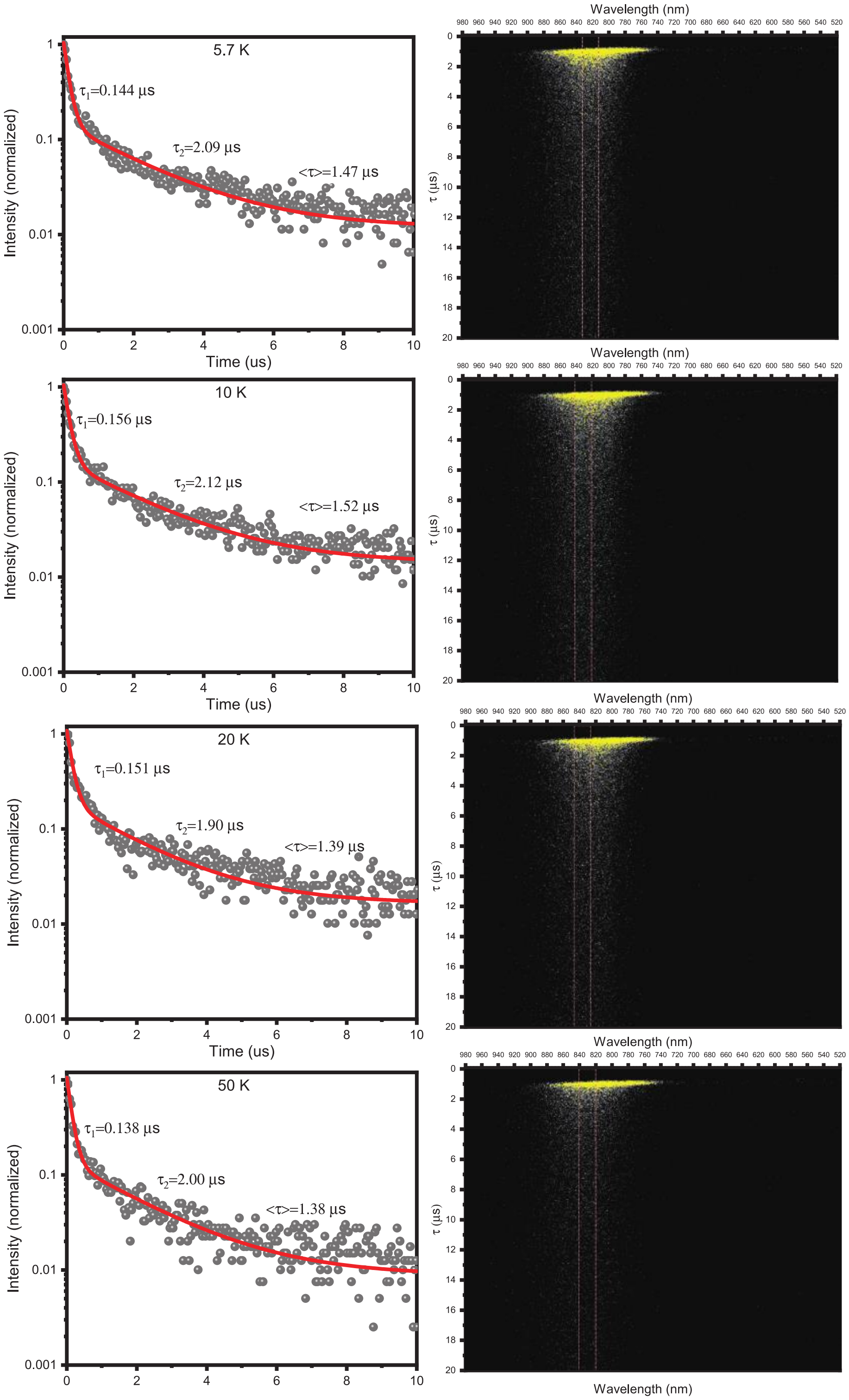

2%Nd:MAPbI₃, 0.11uJ/cm²

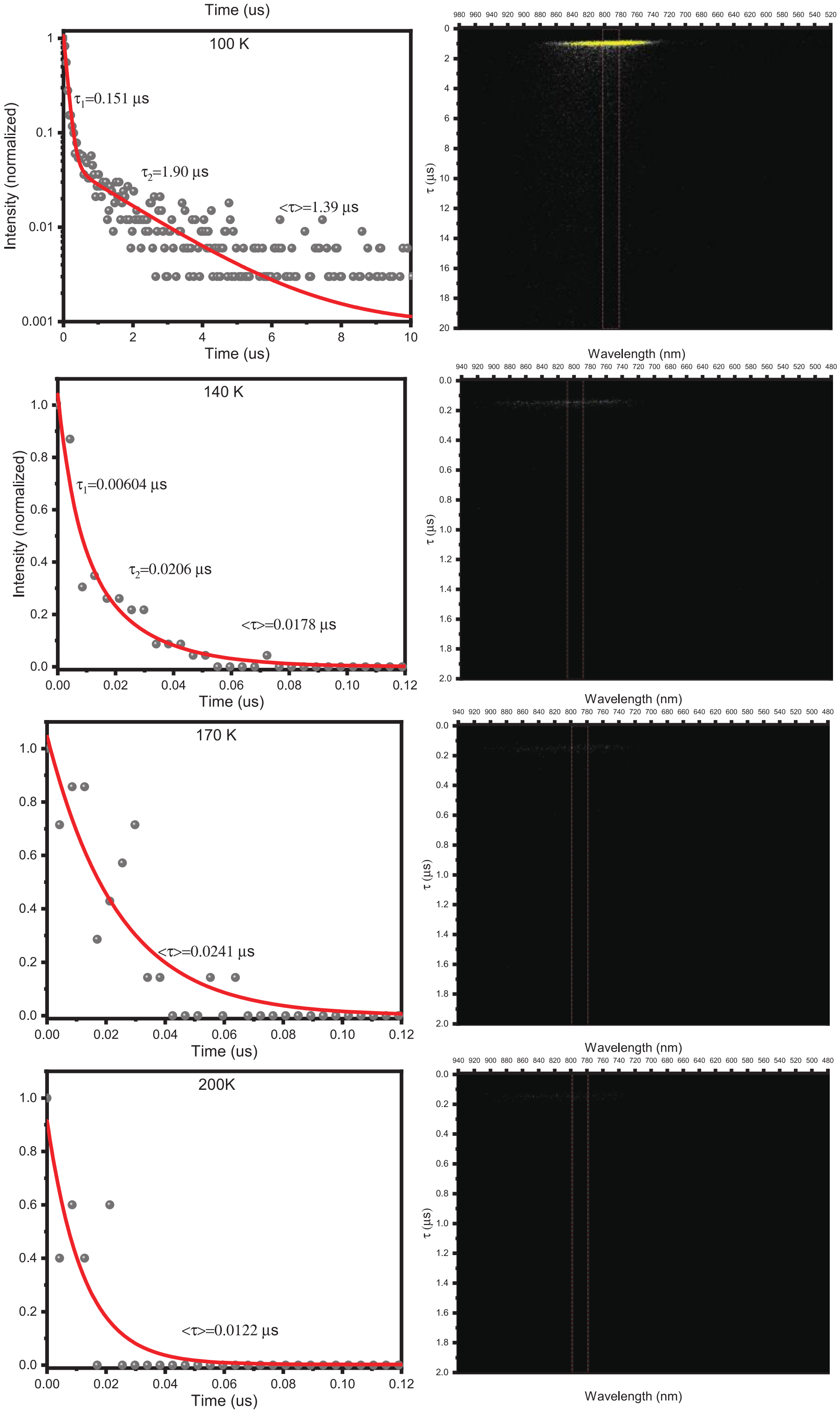

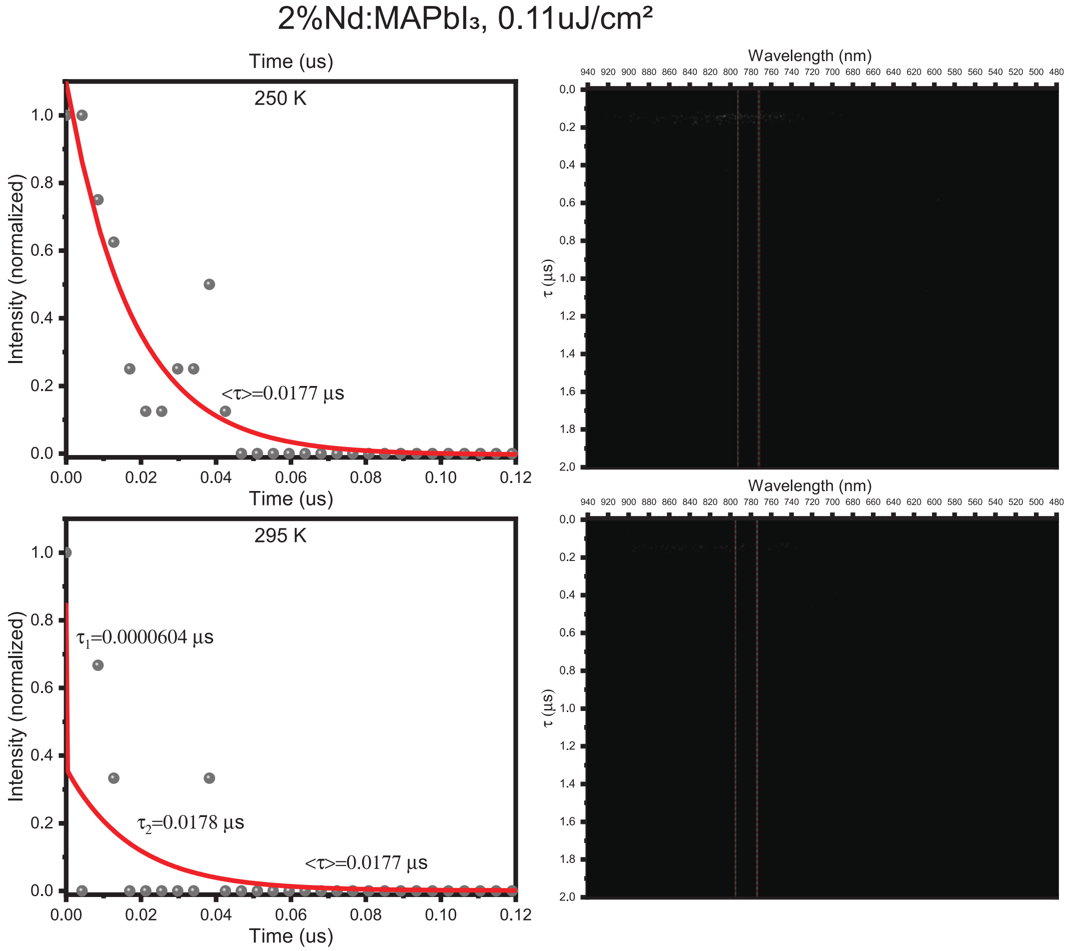

Supplementary Fig. 14. Original time-resolved PL decays of 2%Nd:$MAPbI_3$ (i.e. 2% Nd(II) doping) acquired at 5.7 K – 295 K by streak camera and the fitting details. The fluence — 0.11 μJ/cm^2 is equivalent to 220 μW/cm^2.

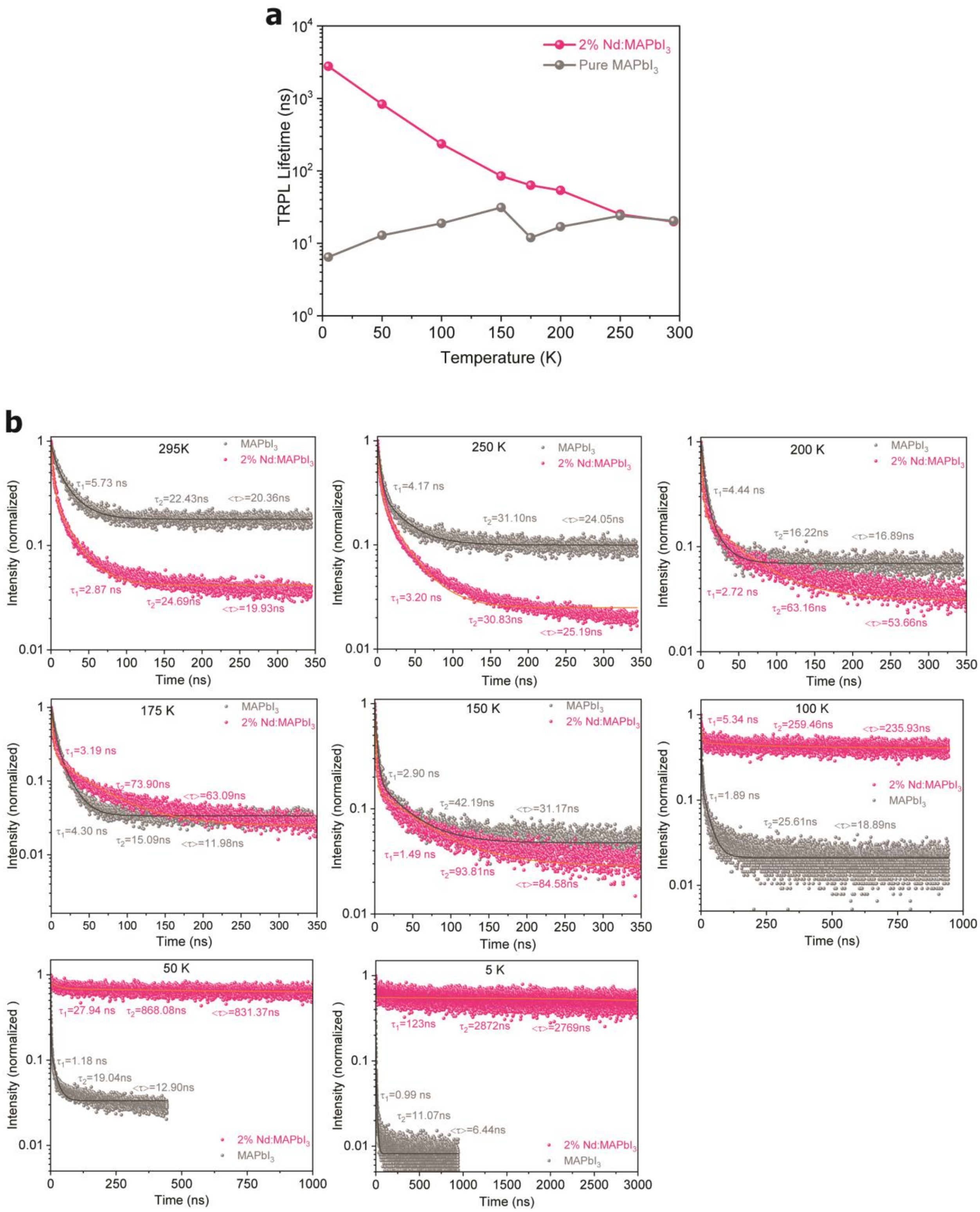

Supplementary Fig. 15. Carrier lifetime and fitting detail by using a laser diode as excitation. **a**, Comparison of lifetime < τ > of peak PL vs temperature for pristine $MAPbI_3$ and 2%Nd:$MAPbI_3$ films. **b**, Associated original data and fitting results.

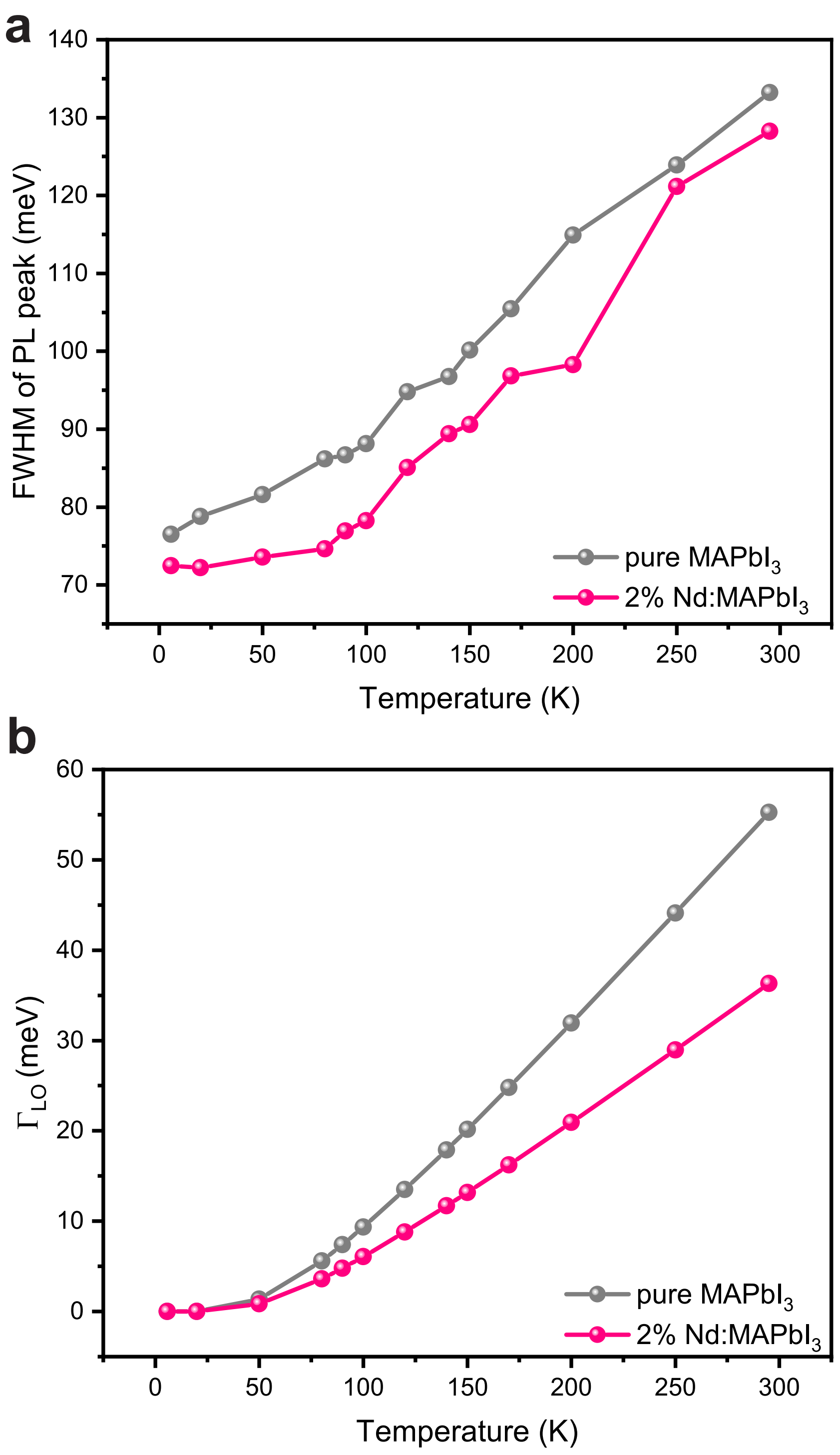

Supplementary Fig. 16. FWHM analysis of the temperature-dependent emission broadening spectra. a, FWHM of PL peak vs temperature for pristine $MAPbI_3$ and 2%Nd:$MAPbI_3$ films. **b**, LO phonon (Fröhlich) scattering vs temperature for pristine $MAPbI_3$ and 2%Nd:$MAPbI_3$ films.

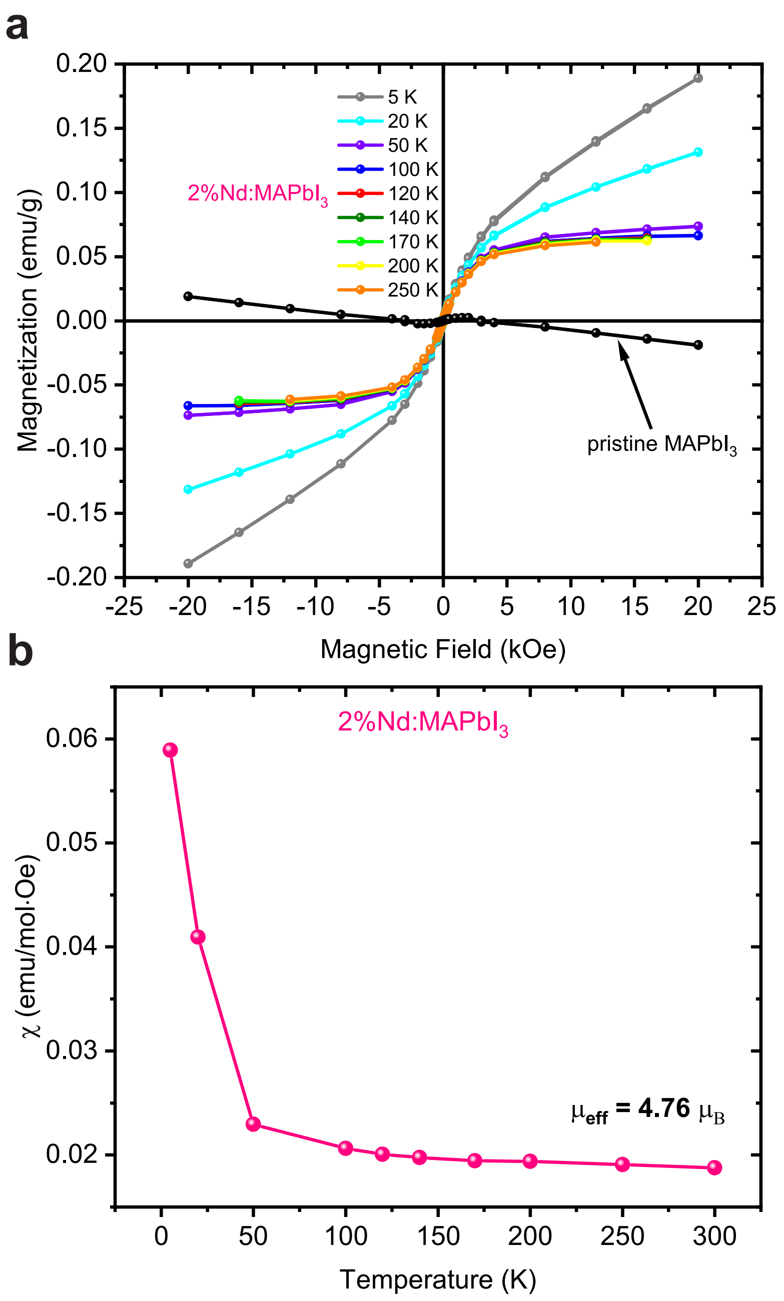

Supplementary Fig. 17. Temperature-dependent magnetization measurements a, Paramagnetic properties of 2%Nd:$MAPbI_3$ powder at different temperatures and diamagnetic properties for pristine $MAPbI_3$ at 300 K. **b,** Temperature-dependent magnetic susceptibility of 2%Nd:$MAPbI_3$ powder.

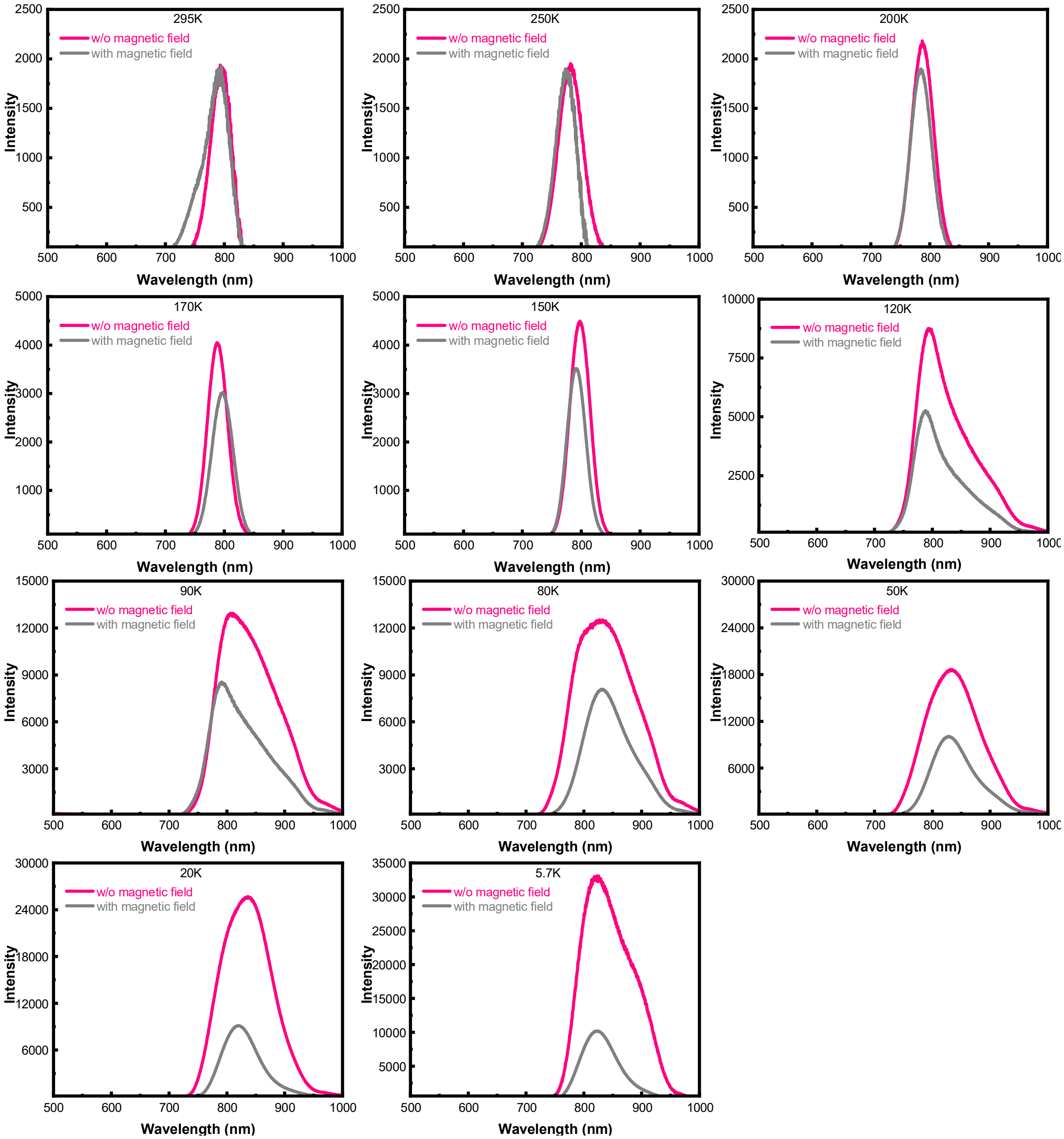

Supplementary Fig. 18. Comparison of ss-PL spectra of 2%Nd:$MAPbI_3$ film without magnetic field (pink) and with the magnetic field (gray) at various temperatures on the same sample.

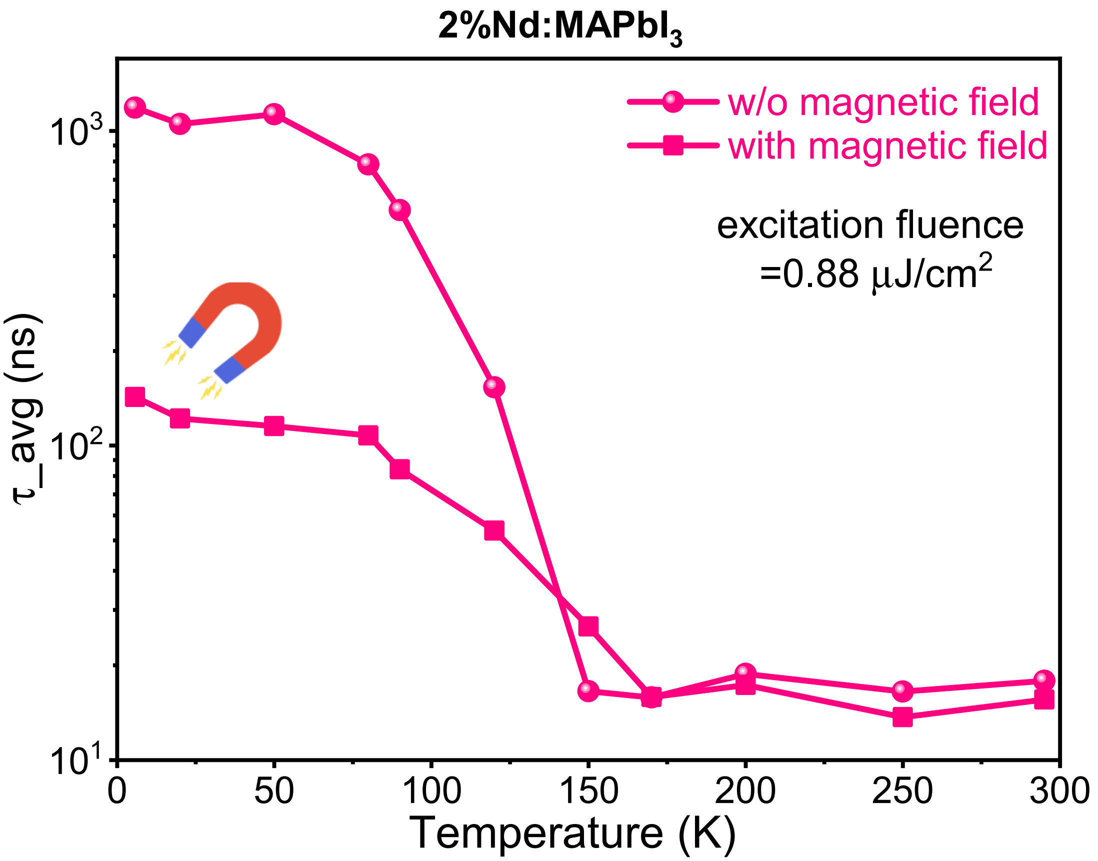

Supplementary Fig. 19. Temperature-dependent average PL lifetimes of 2%Nd:$MAPbI_3$ film under magnetic field (pink line with square dot) and without the magnetic field (pink line with round dot). A SmCo magnet was applied on the copper cold finger behind the sample, and the system is vacuumed and cooled down to 5K first. The PL spectra was acquired at each set temperature during warming up process from 5K to room temperature. Then, the magnet was removed and the same sample was vacuumed and cooled down again to 5K. The PL spectra at each set temperature without magnetic field was acquired during warming up process from 5K to room temperature.

pristine $MAPbI_3$, 0.88uJ/cm²

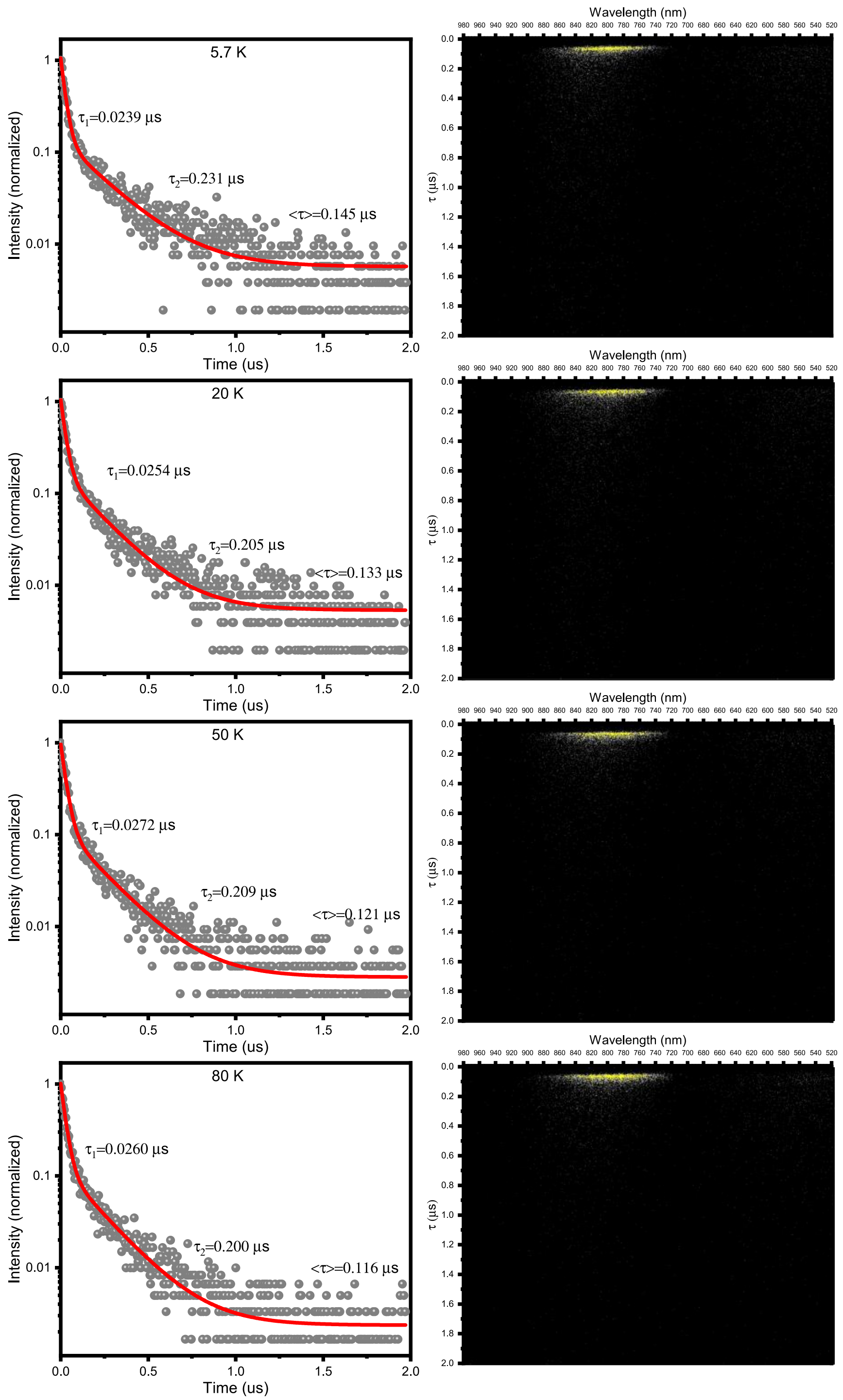

pristine $MAPbI_3$, 0.88uJ/cm²

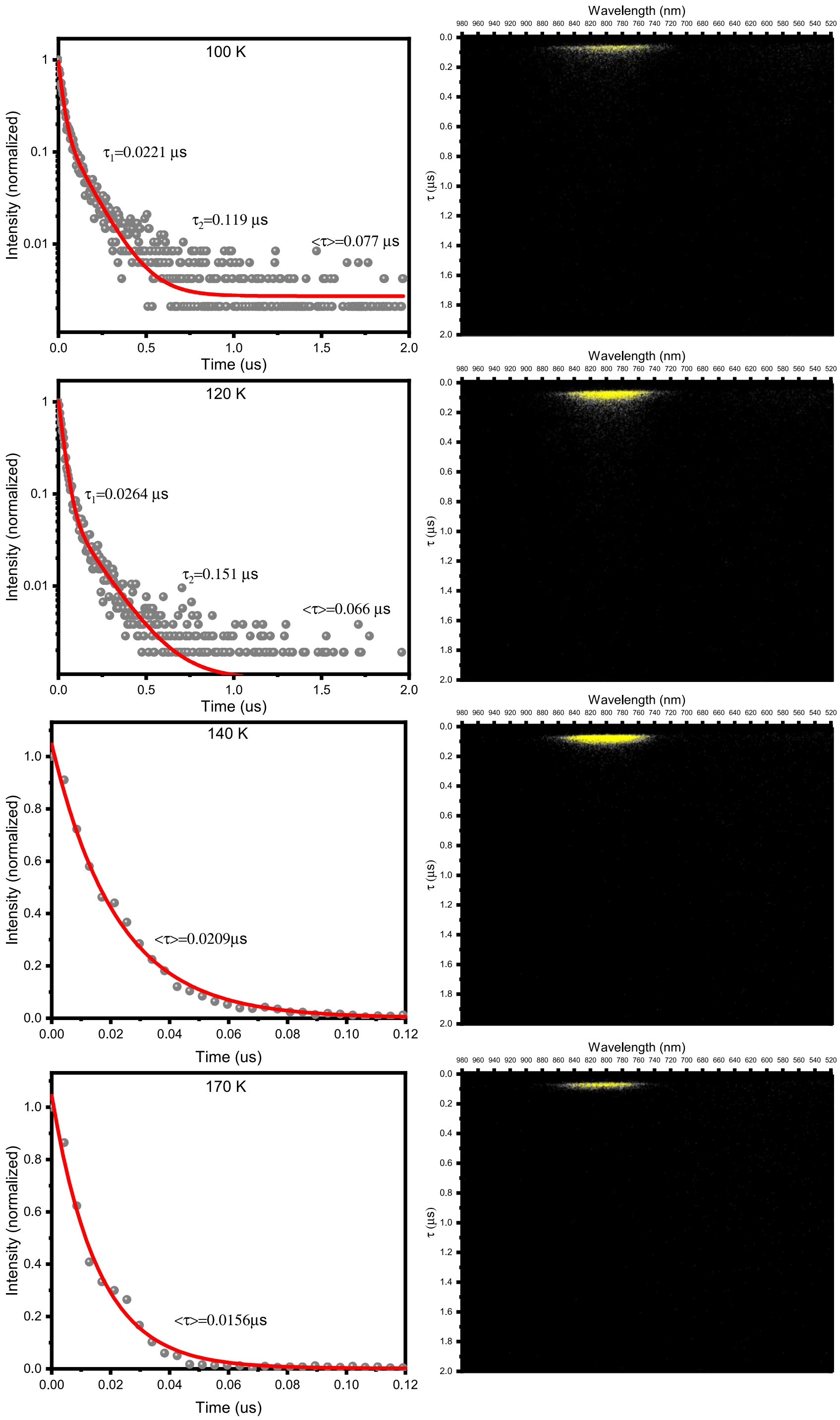

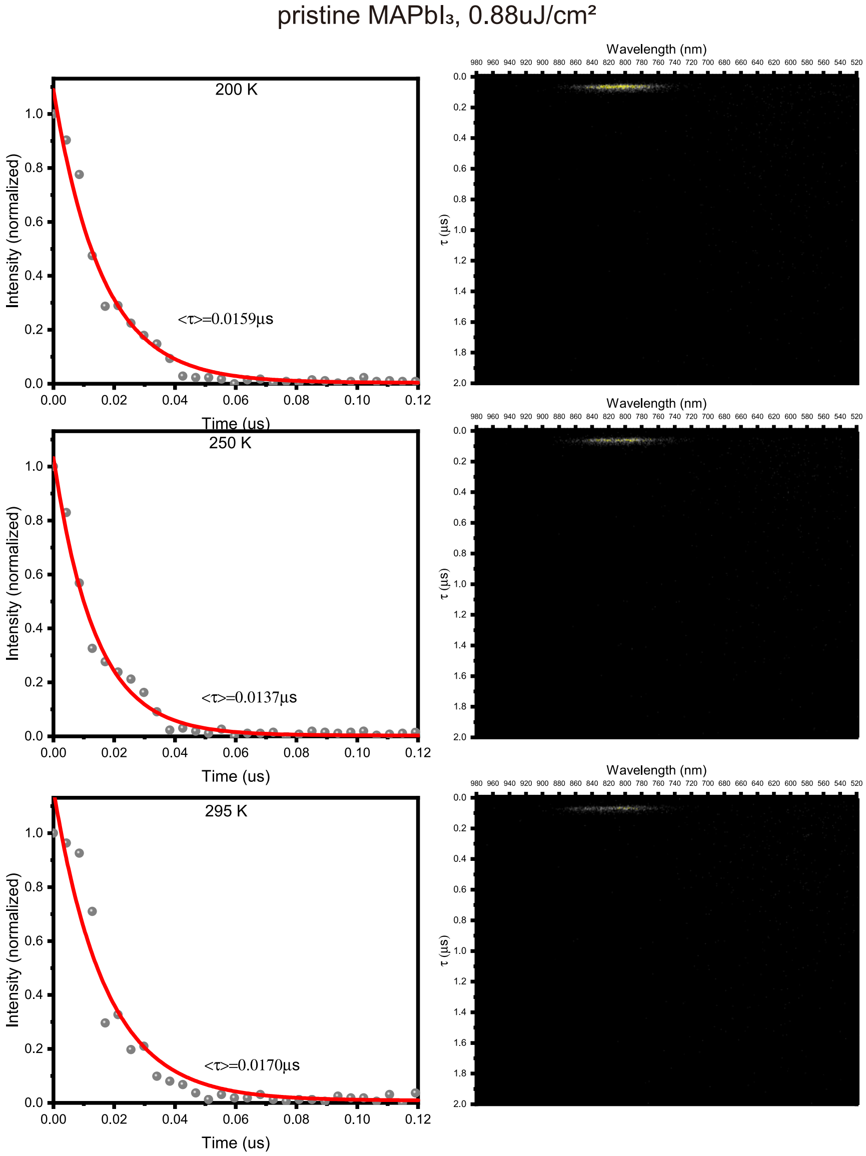

Supplementary Fig. 20. Original time-resolved PL decays of pristine $MAPbI_3$ (i.e. 0% Nd(II) doping) acquired at 5.7 K – 295 K by streak camera and the fitting details under magnetic field strength of 1500 Gauss. Excitation is 400-nm ultrafast laser with pulse duration 35 fs with fluence—0.88 μJ/cm^2, equivalent to 1760 μW/cm^2.

2%Nd:$MAPbI_3$, 0.88uJ/cm²

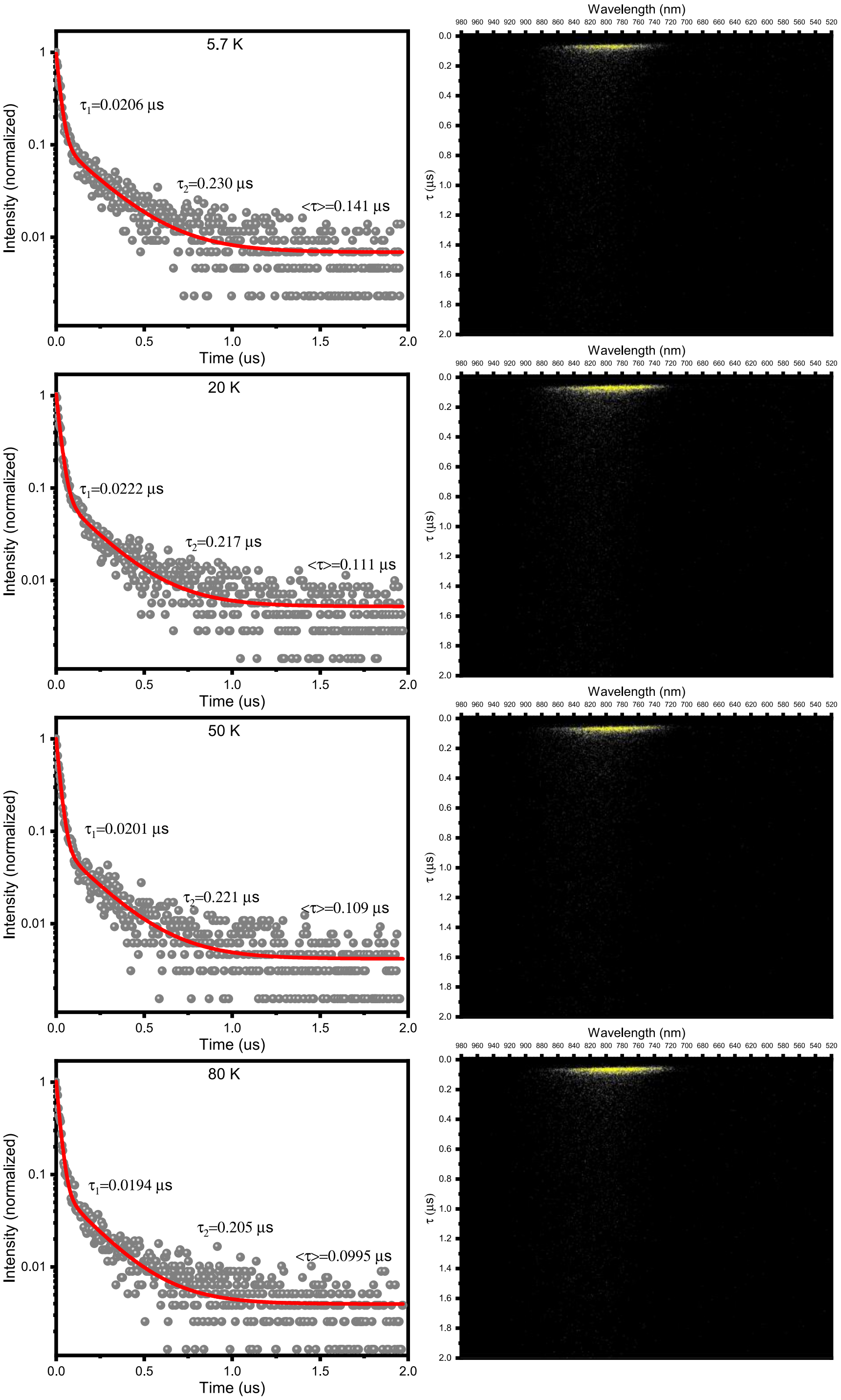

2%Nd:$MAPbI_3$, 0.88uJ/cm²

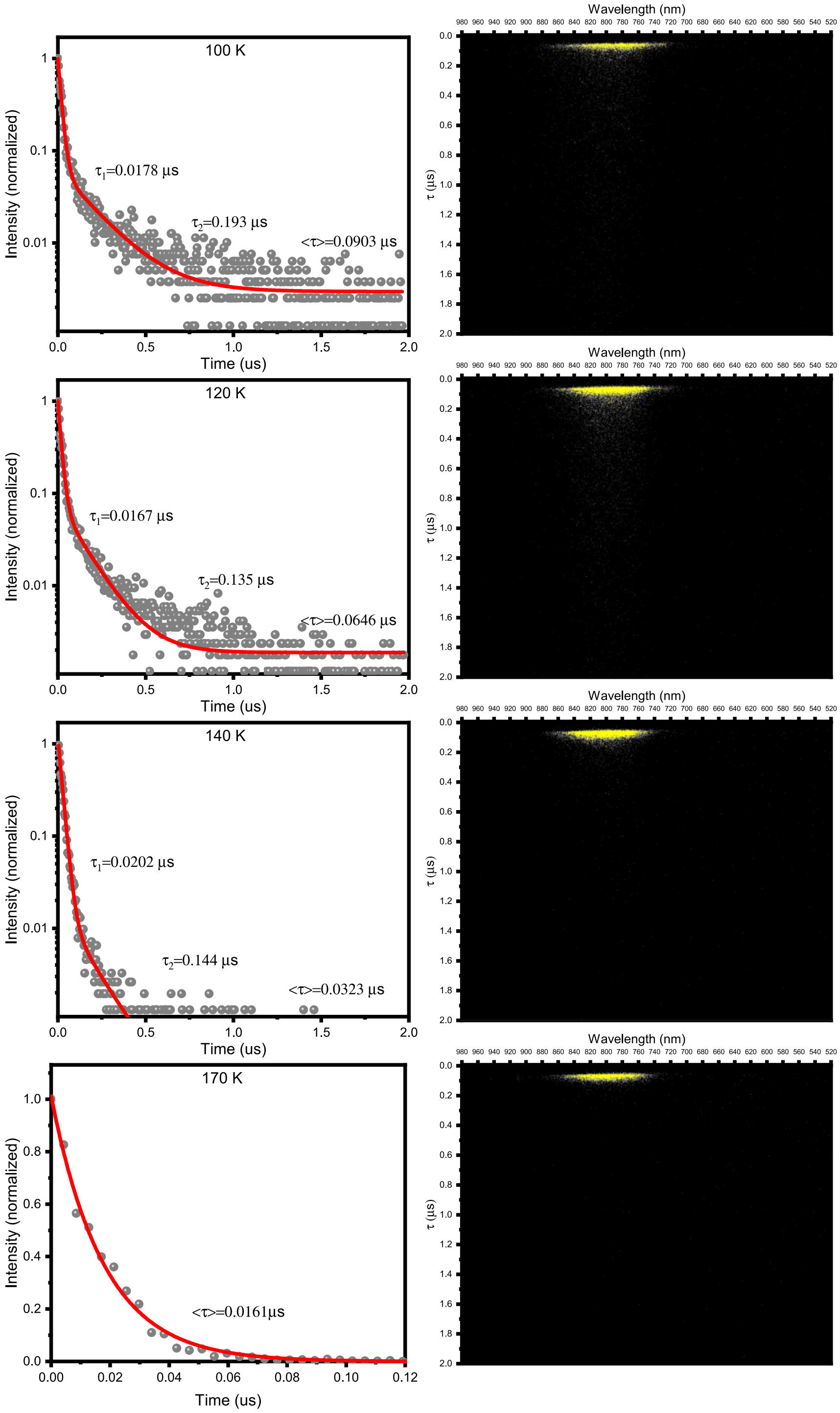

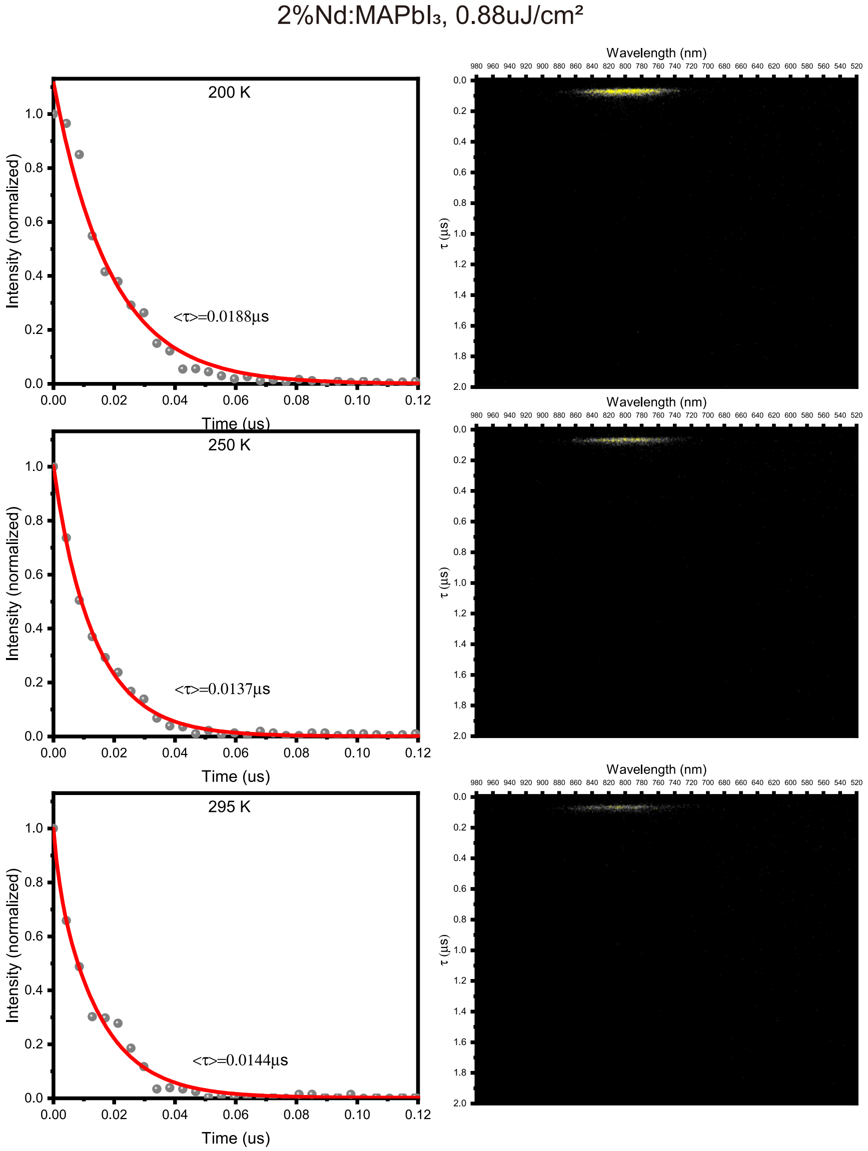

Supplementary Fig. 21. Original time-resolved PL decays of 2%Nd:$MAPbI_3$ (i.e. 2% Nd(II) doping) acquired at 5.7 K-295 K by streak camera and the fitting details under magnetic field strength of 1500 Gauss. Excitation is 400-nm ultrafast laser with pulse duration 35 fs with fluence—0.88 μJ/cm^2, equivalent to 1760 μW/cm^2.

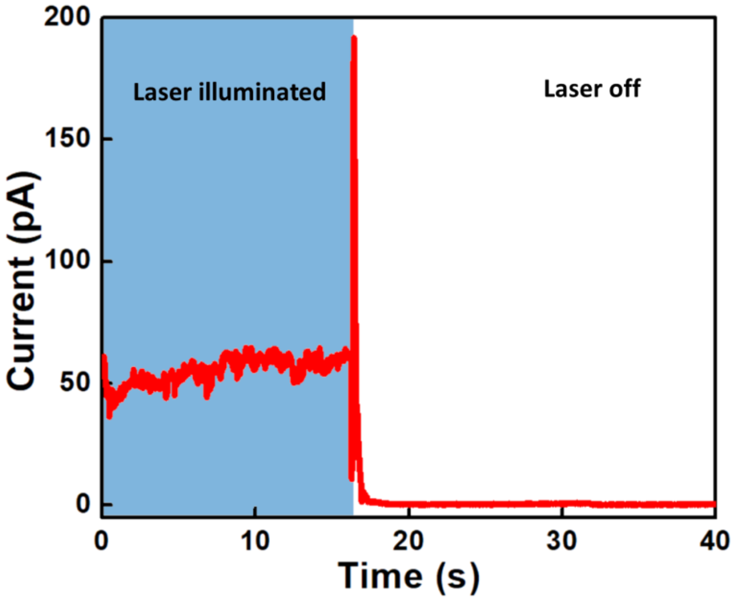

Supplementary Fig. 22. The tunneling current under 400nm laser illumination and under dark conditions. The STM tip was positioned above the sample using 1 V, 50 pA tunneling condition and then held fixed by turning off the constant-current feedback throughout the data collection. In the first ~17 sec of laser illumination, the tunneling current was maintained at 50 pA (the small increment was due to uncompensated mechanical drift due to feedback cut-off). During the dark condition, there was no measurable tunneling current above the pre-amplifier noise.

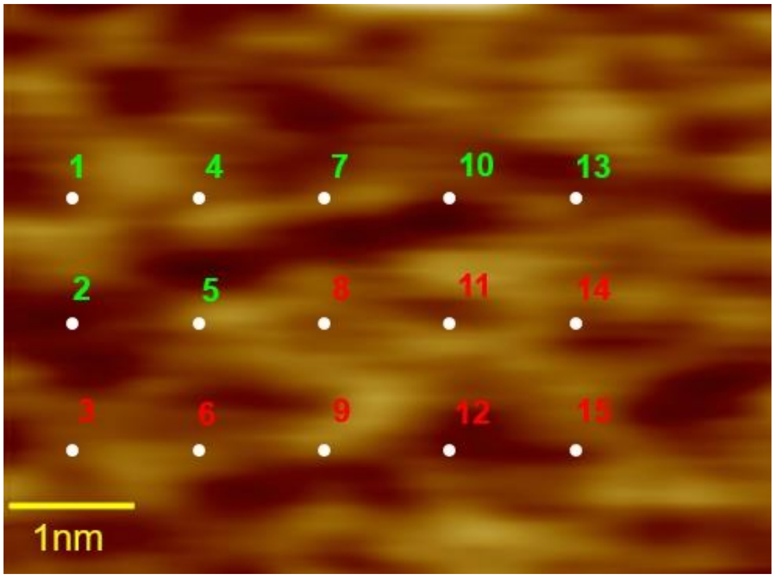

Supplementary Fig. 23. Tunneling spectroscopy of Nd^{2+}-doped $MAPbI_3$ perovskite with local sampling. An STM image of a Nd^{2+} doped sample surface acquired under 400 nm illumination (V_t = 1 V, I_t = 50 pA).

Supplementary Fig. 23 shows an STM image of a Nd^{2+}-doped $MAPbI_3$ perovskite sample. Here, the distances between the grid points are 1 nm x 1 nm. The tunneling spectroscopy data were recorded under 400 nm illumination. The green number locations give a semiconducting gap (~1.5 V), while the red number locations exhibit a smaller gap-like feature and show symmetric peaks in d^2I/dV^2 data.

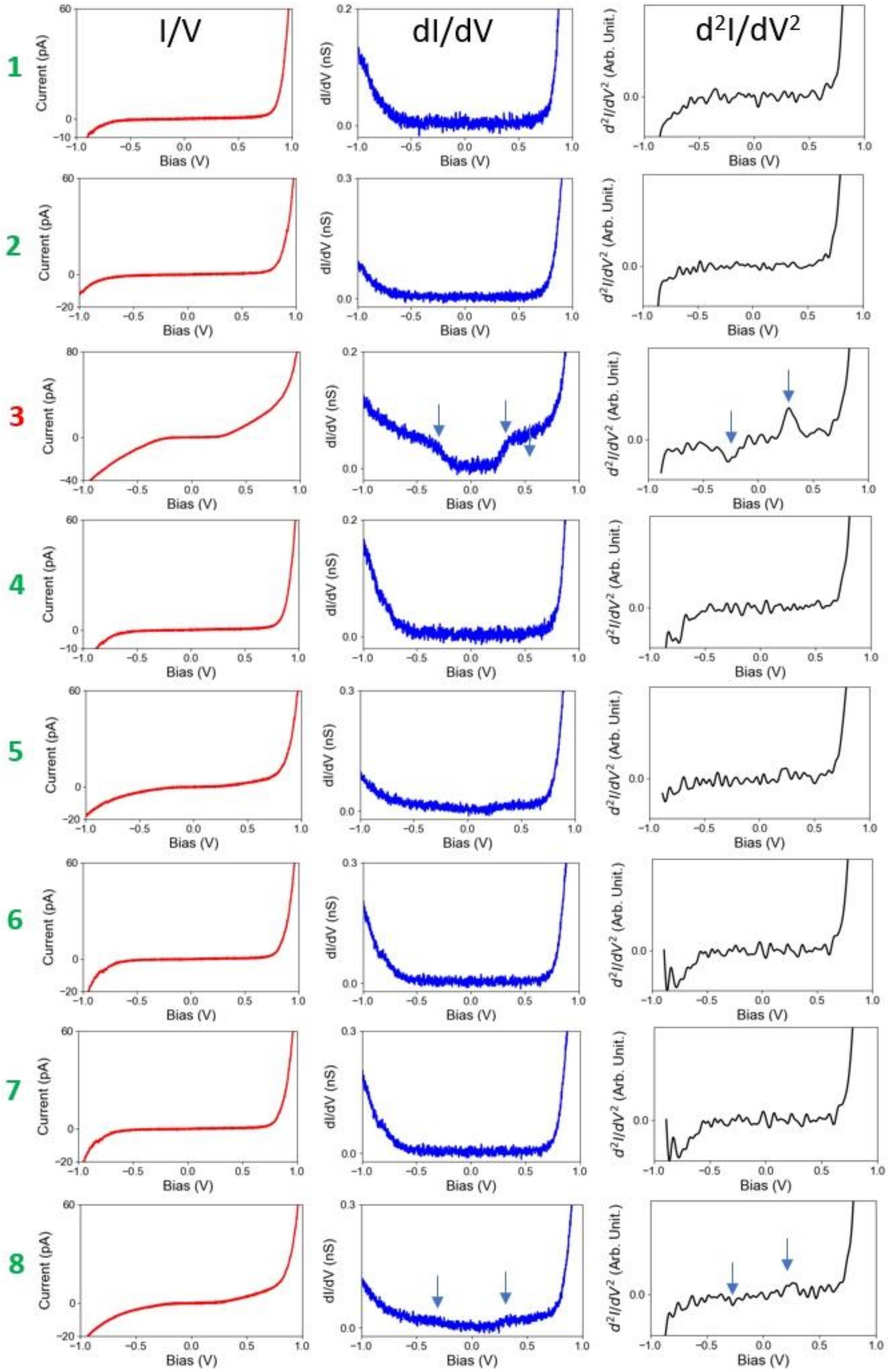
I/V
dI/dV
d2I/dV2
1
2
3
4
5
6
7
8
Current (pA)
dI/dV (nS)
d2I/dV2 (Arb. Unit.)
Bias (V)

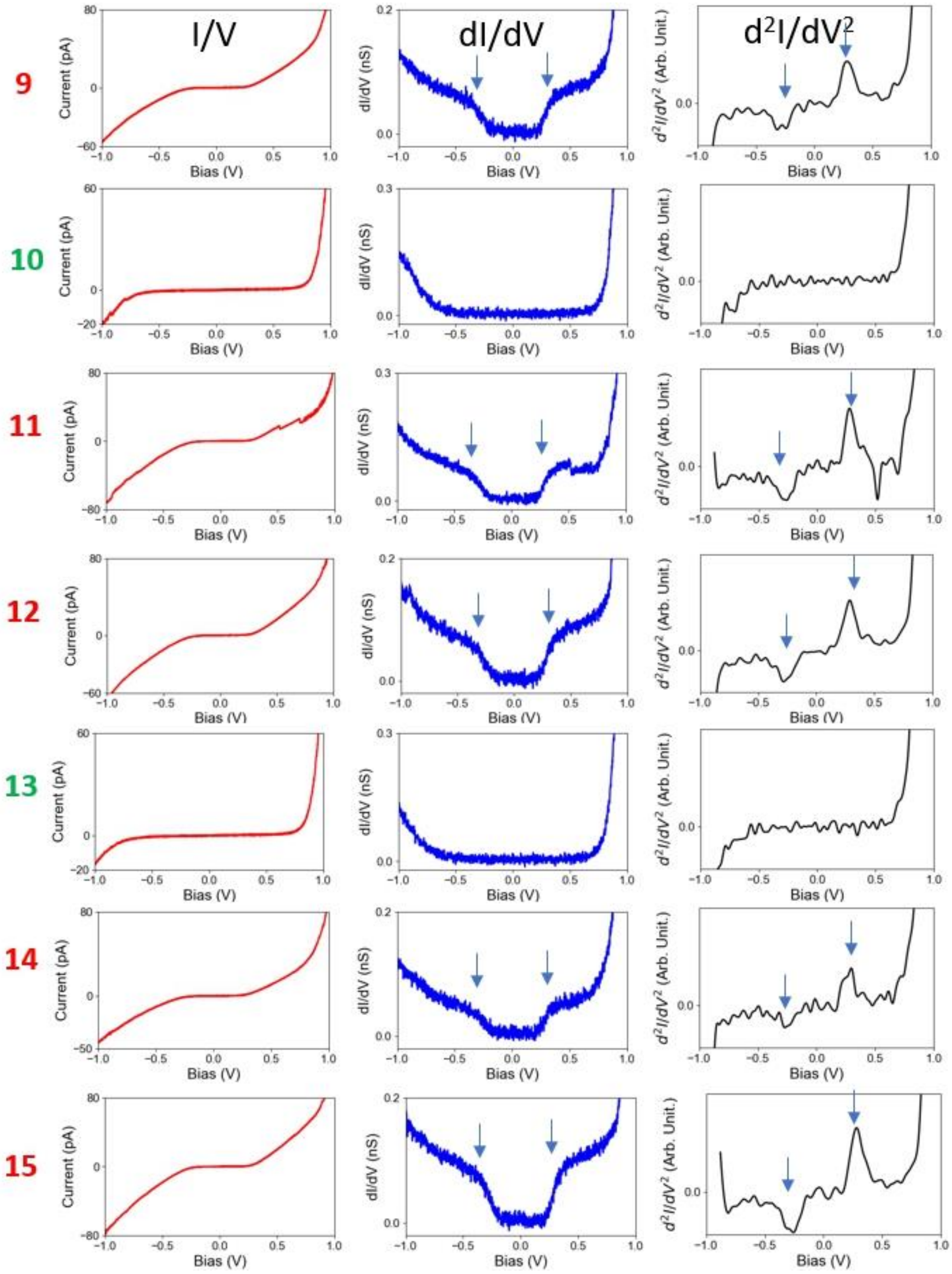

Supplementary Fig. 24. I-V (left), dI/dV (middle), and d^2I/dV^2 (right) curves of Nd^{2+}-doped $MAPbI_3$ perovskite with local area sampling. Here, the spectroscopy numbers at the left correspond to the numbers shown in Supplementary Fig. 23. Note that at the location '8', only a small gap-like feature is observed (indicated with arrows).

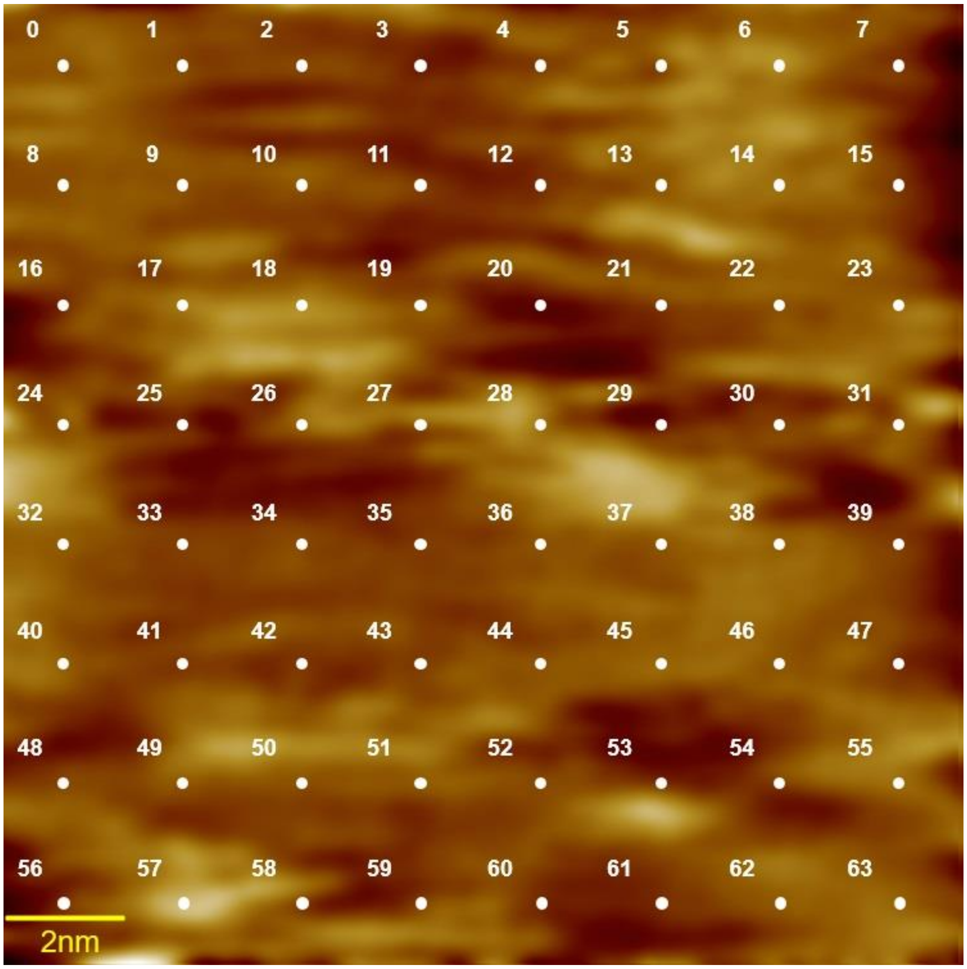

Supplementary Fig. 25. Tunneling spectroscopy of Nd^{2+}-doped $MAPbI_3$ perovskite with spatially periodic sampling. An STM image of a Nd^{2+}-doped sample surface acquired under 400 nm illumination (V_t = 1 V, I_t = 50 pA).

Supplementary Fig. 25 shows an STM image of a Nd^{2+}-doped $MAPbI_3$ perovskite sample. Here, the distances between the grid points are 2 nm x 2 nm. The tunneling spectroscopy data were recorded under 400 nm illumination, and all the spectroscopy recorded at this location exhibited a gap-like feature (see the following spectroscopic data).

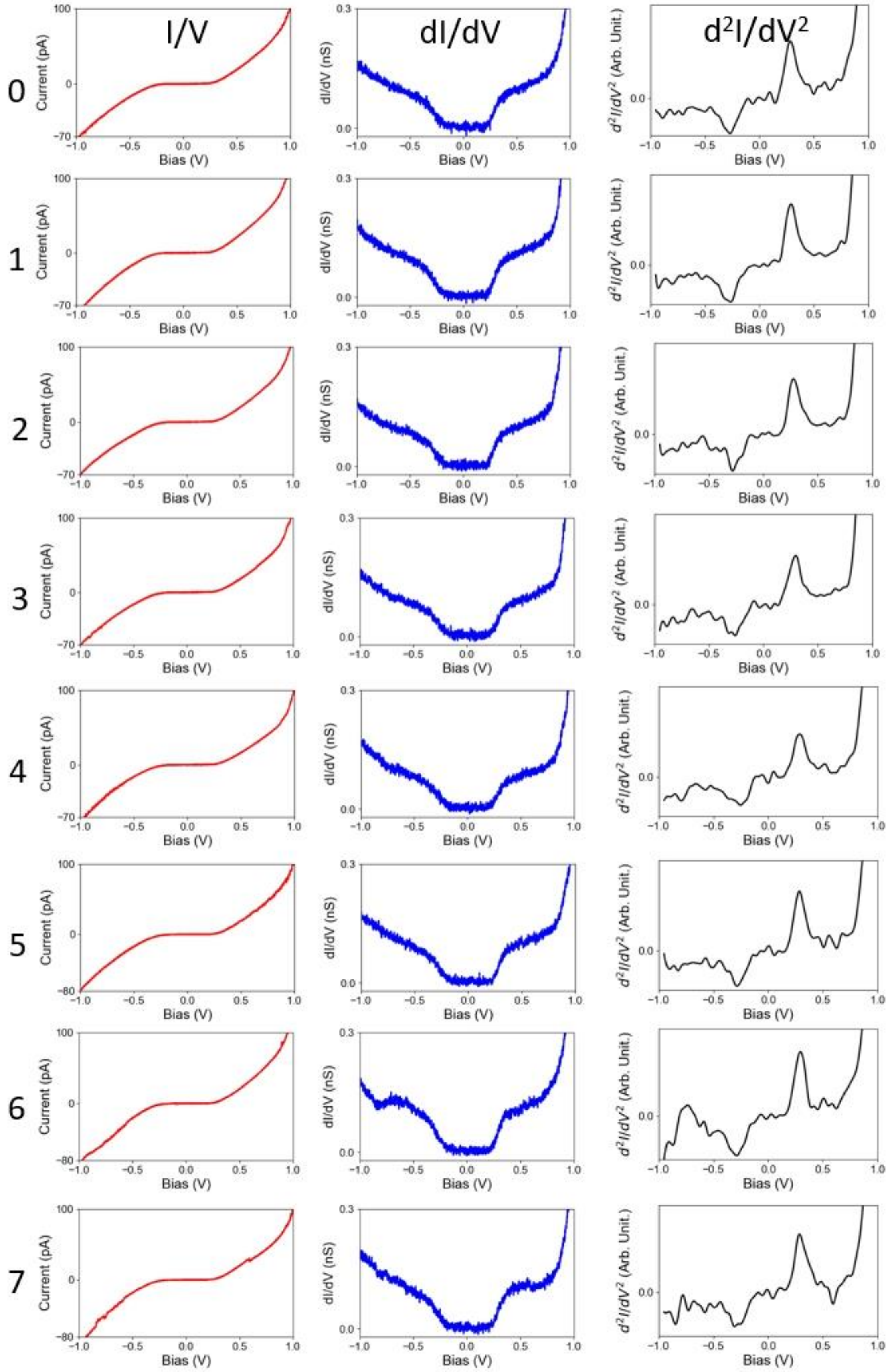

I/V
dI/dV
d²I/dV²
0
1
2
3
4
5
6
7
Current (pA)
dI/dV (nS)
d²I/dV² (Arb. Unit.)
Bias (V)

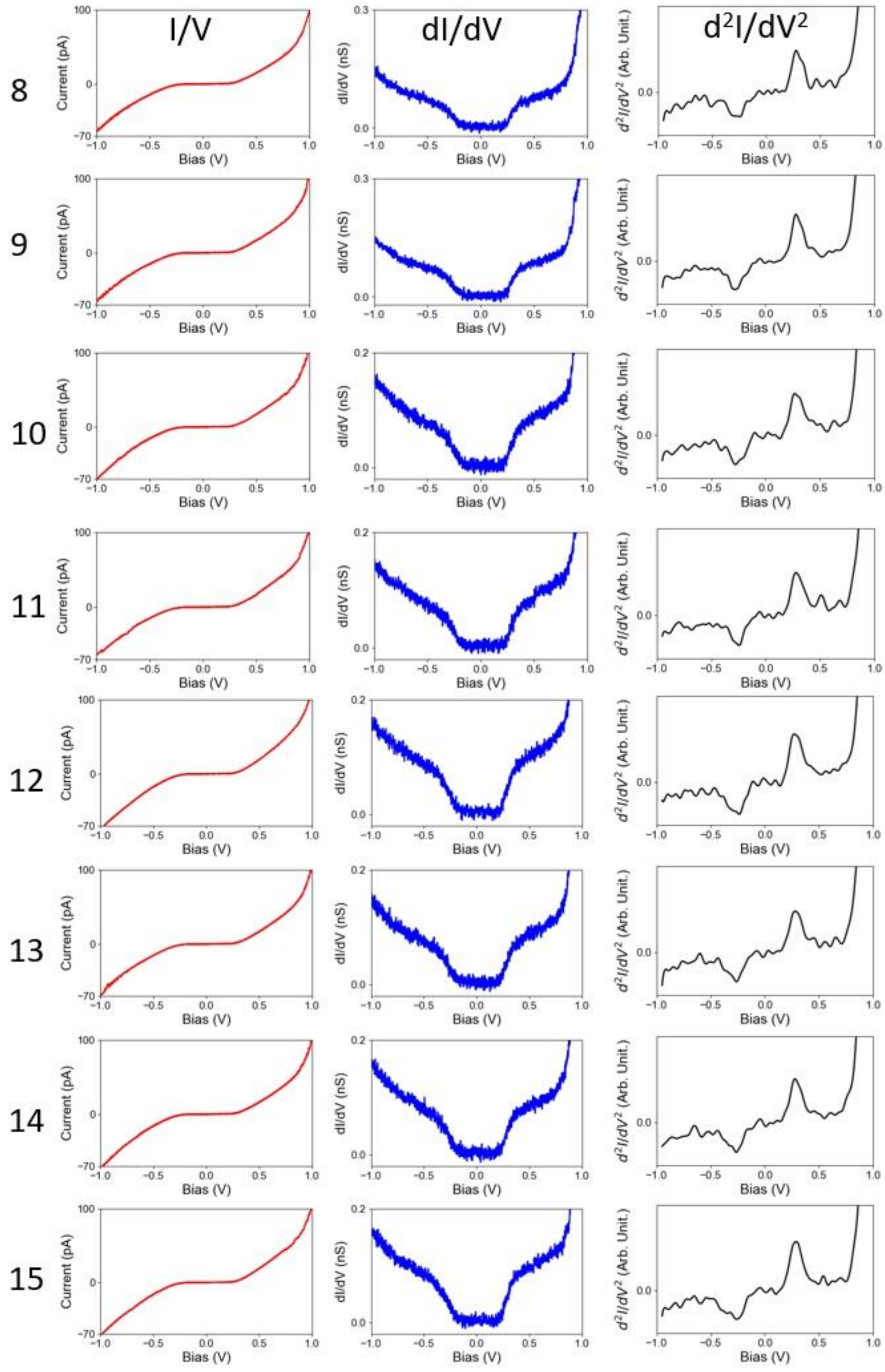
I/V
dI/dV
d^2I/dV^2
8
9
10
11
12
13
14
15
Current (pA)
dI/dV (nS)
d^2I/dV^2 (Arb. Unit.)
Bias (V)

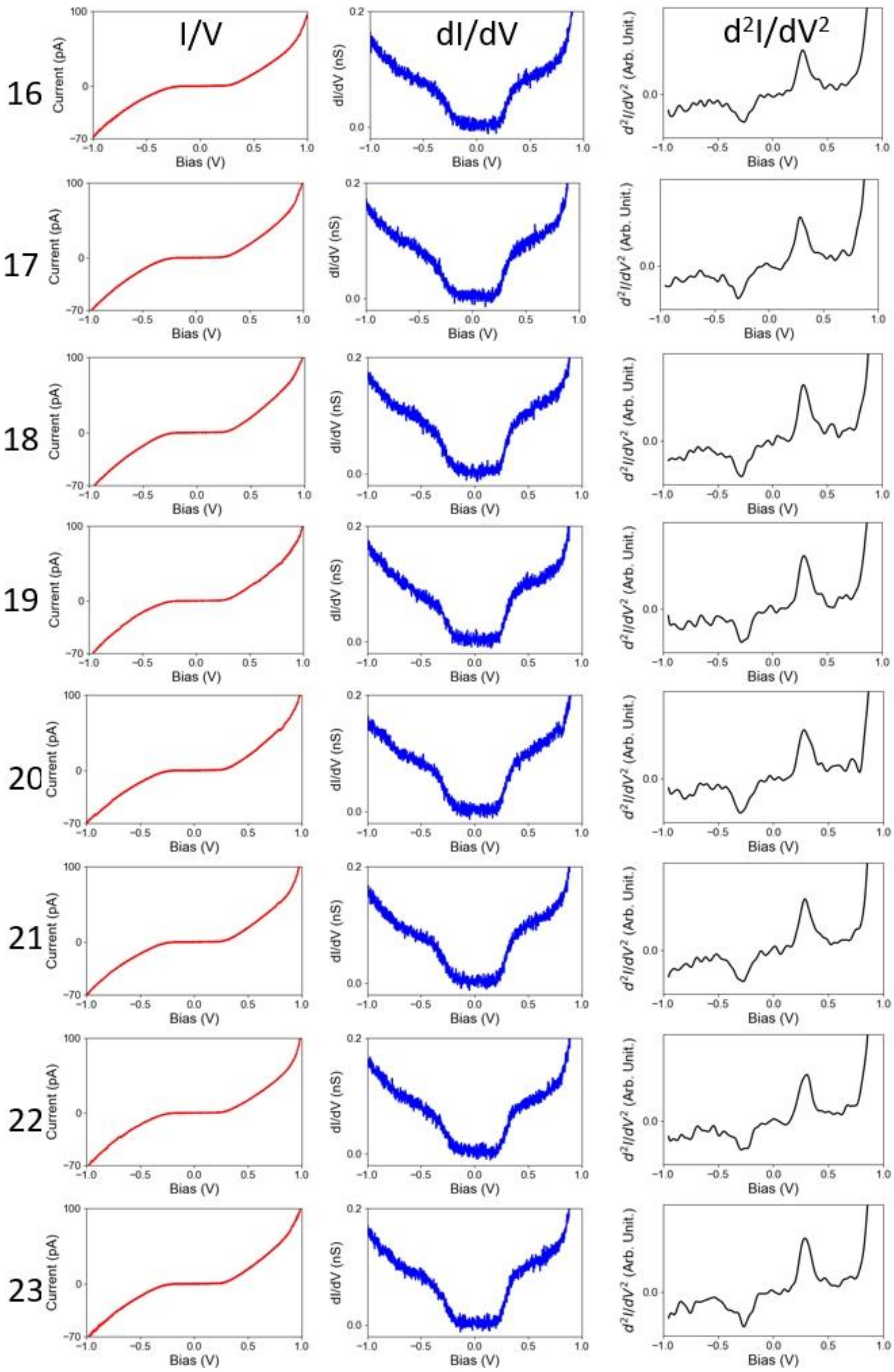
I/V
dI/dV
d2I/dV2
16
17
18
19
20
21
22
23
Current (pA)
Bias (V)
dI/dV (nS)
d2I/dV2 (Arb. Unit.)

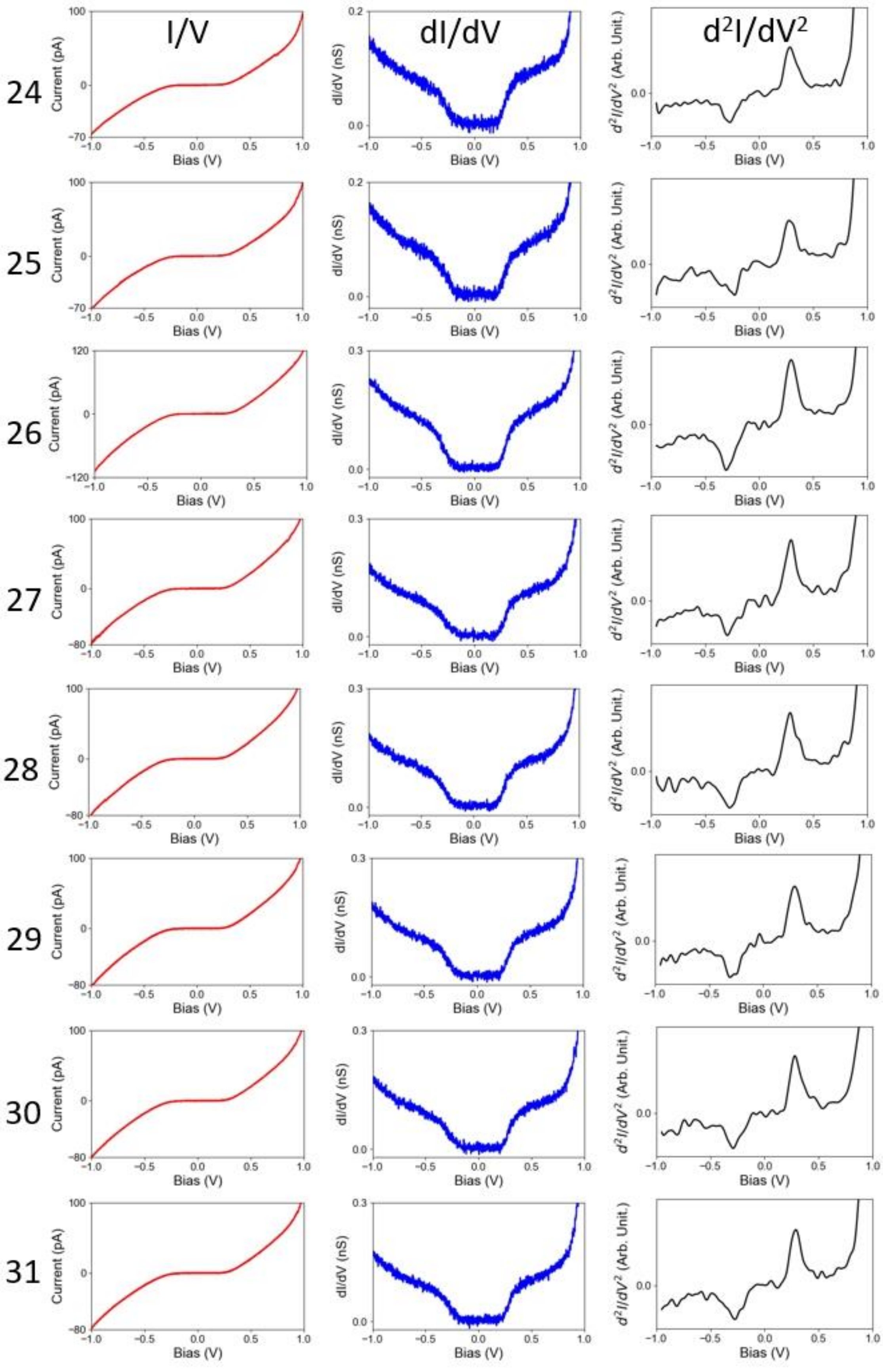
I/V
dI/dV
d2I/dV2
24
25
26
27
28
29
30
31
Current (pA)
dI/dV (nS)
d2I/dV2 (Arb. Unit.)
Bias (V)

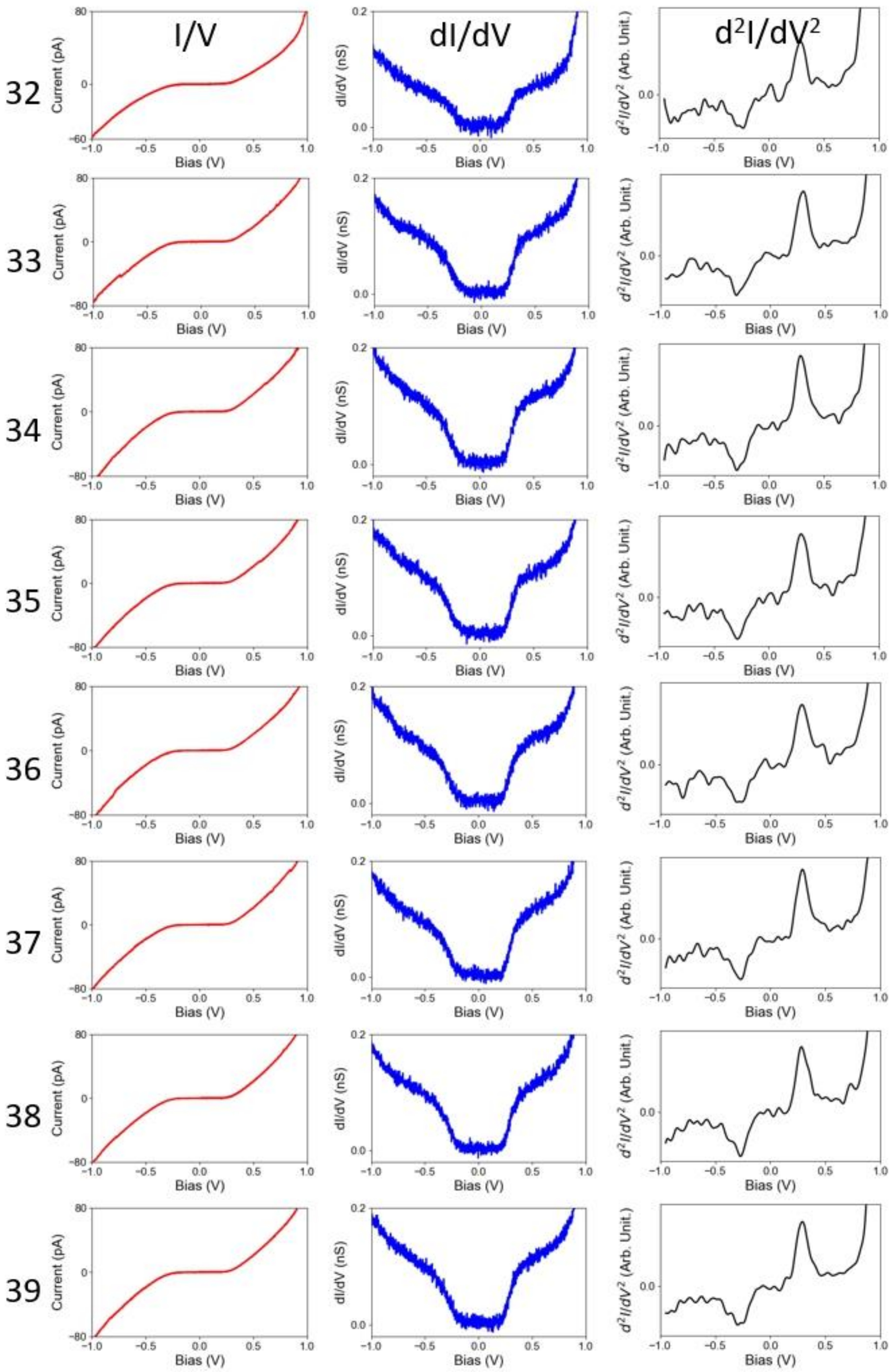
I/V
dI/dV
d2I/dV2
32
33
34
35
36
37
38
39
Current (pA)
dI/dV (nS)
d2I/dV2 (Arb. Unit.)
Bias (V)

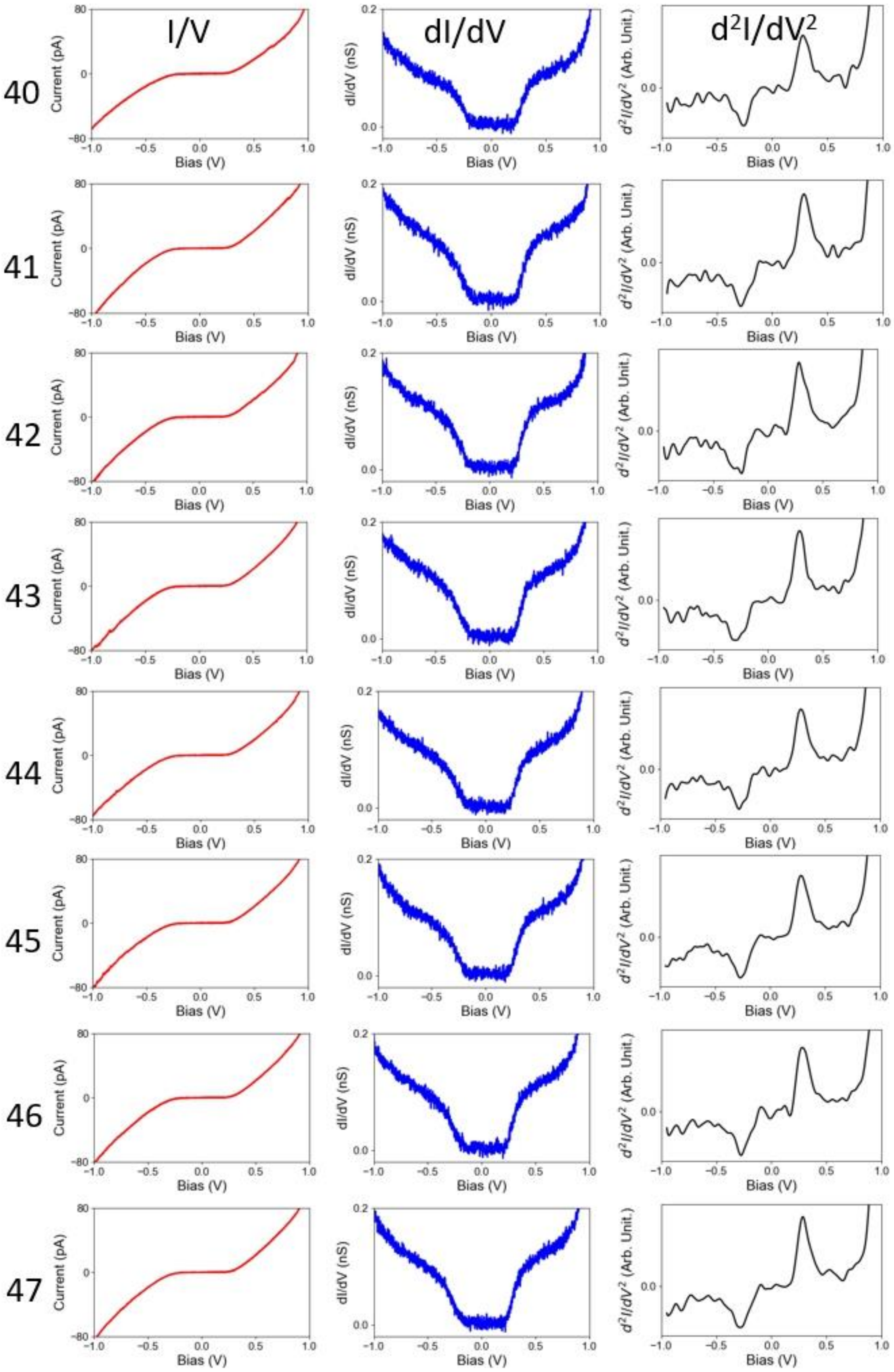
I/V
dI/dV
d2I/dV2
40
41
42
43
44
45
46
47
Current (pA)
dI/dV (nS)
d2I/dV2 (Arb. Unit.)
Bias (V)

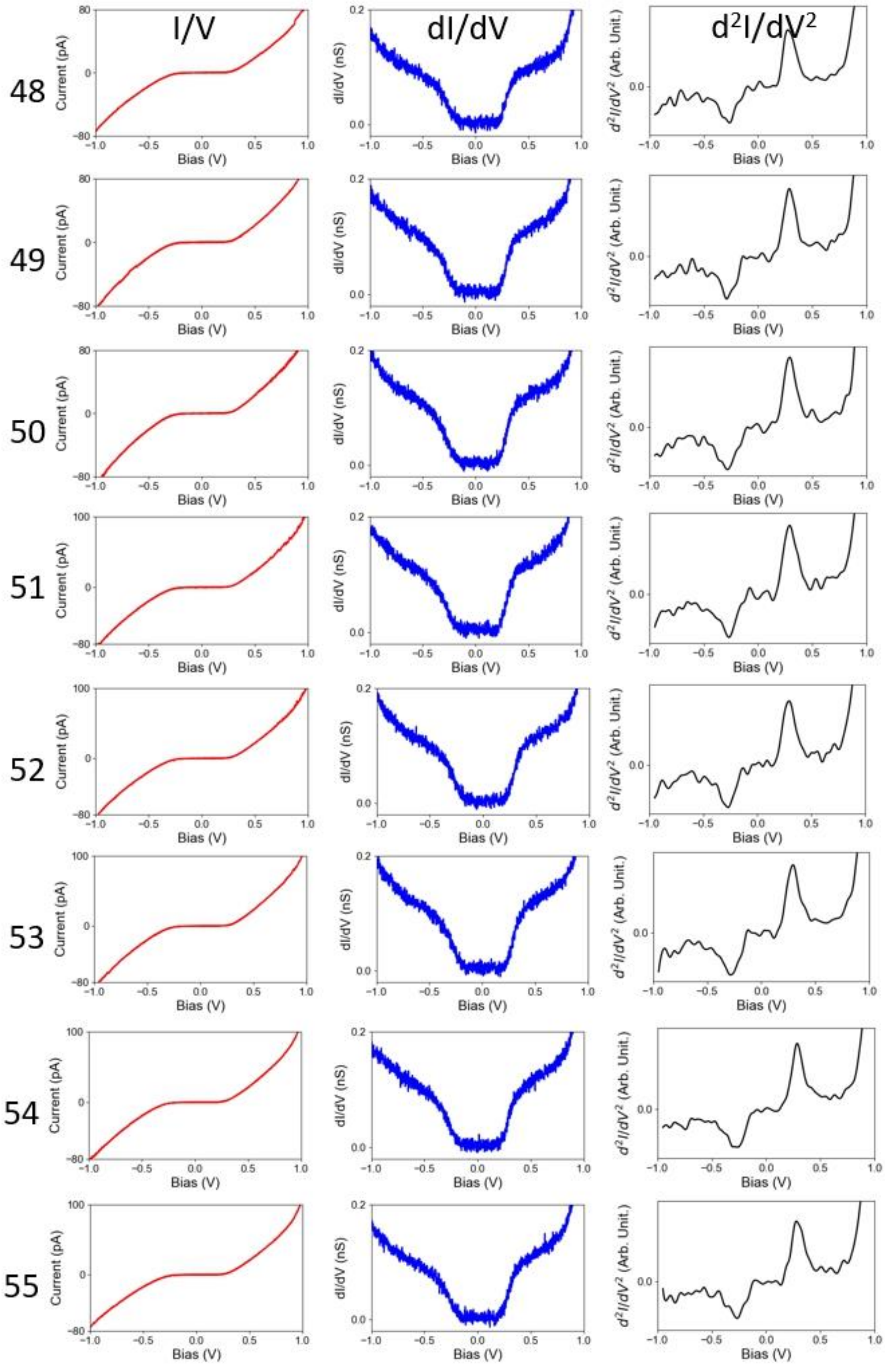
I/V
dI/dV
d2I/dV2
48
49
50
51
52
53
54
55
Current (pA)
dI/dV (nS)
d2I/dV2 (Arb. Unit.)
Bias (V)

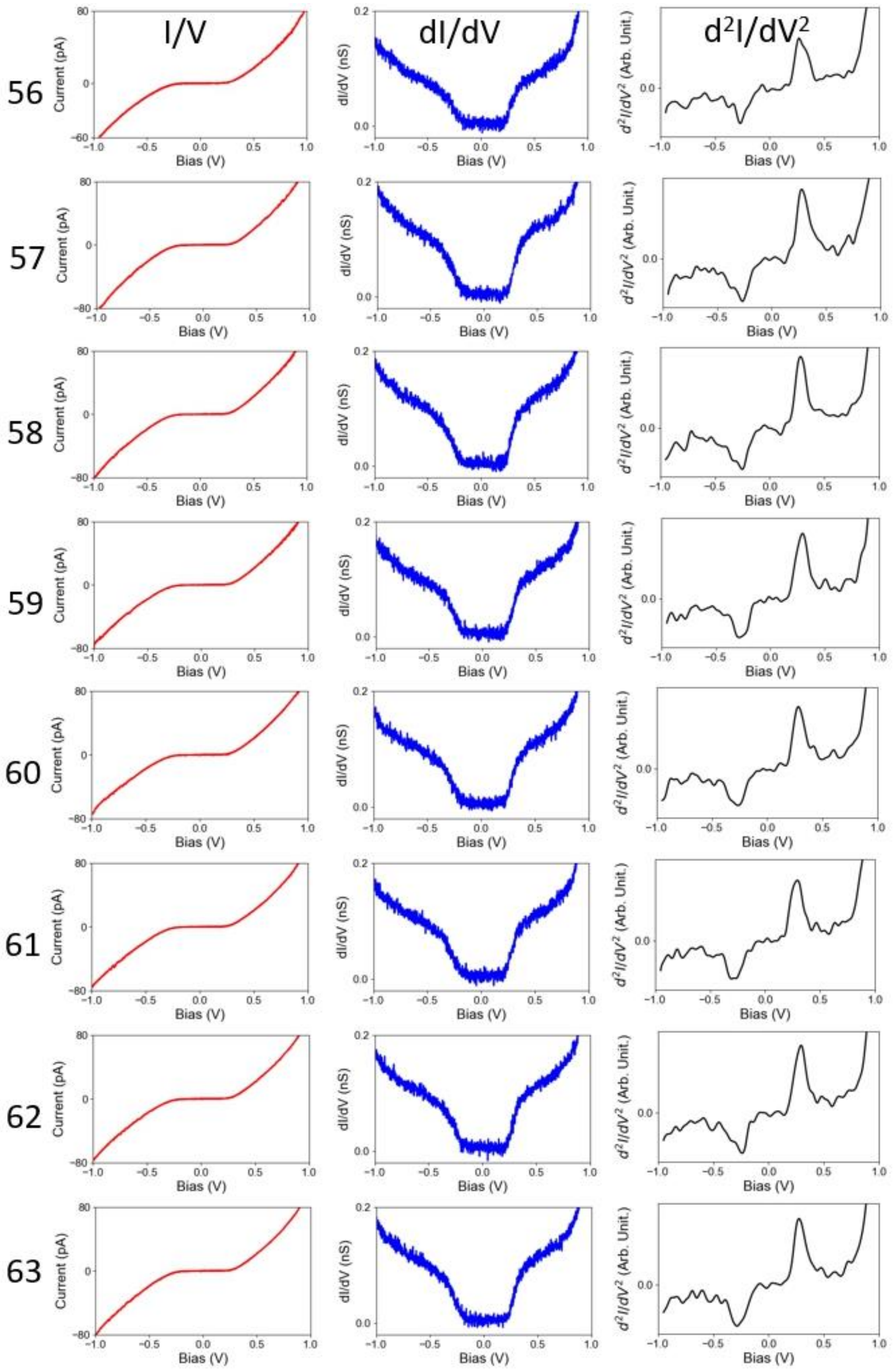

Supplementary Fig. 26. I-V (left), dI/dV (middle), and d^2I/dV^2 (right) curves of Nd^{2+}-doped $MAPbI_3$ perovskite with spatially periodic sampling. Here, the spectroscopy numbers at the left correspond to the numbers shown in Supplementary Fig. 25.

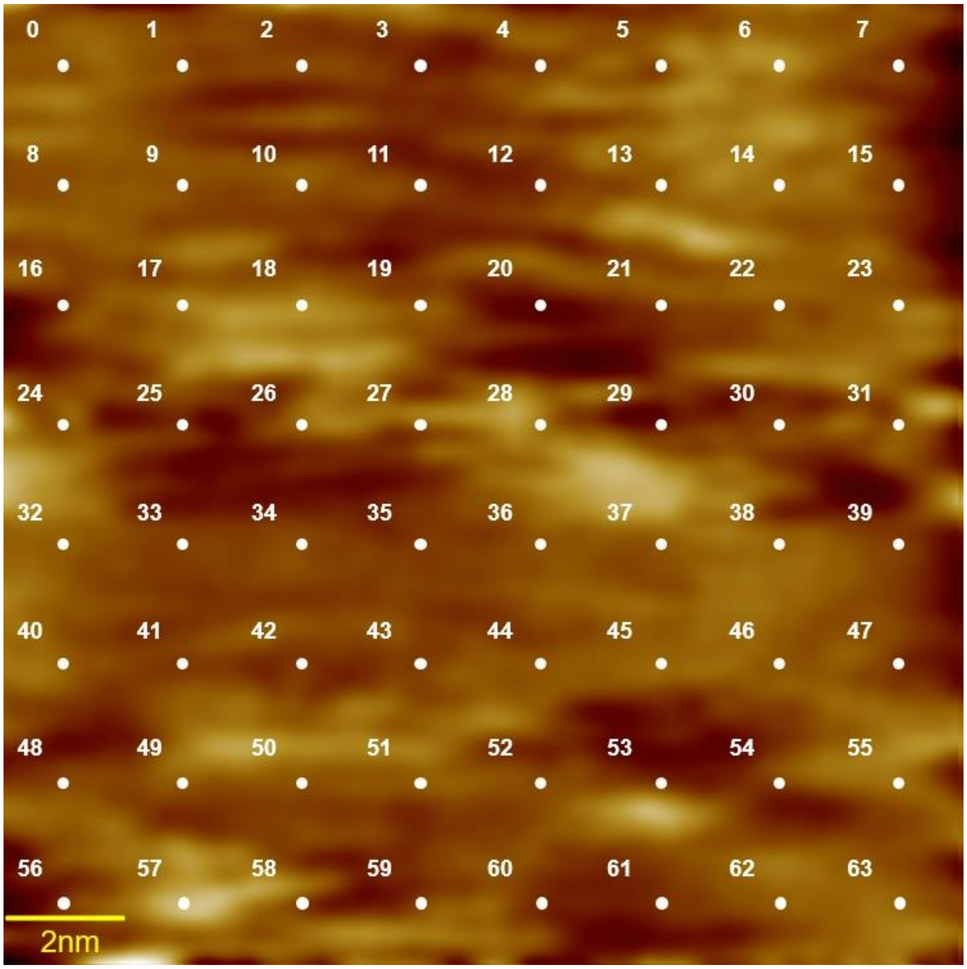

Supplementary Fig. 27. Tunneling spectroscopy of pristine $MAPbI_3$ perovskite with spatially periodic sampling. An STM image of an undoped sample surface acquired under 400 nm illumination (V_t = 1 V, I_t = 50 pA).

Supplementary Fig. 27 shows an STM image of an undoped $MAPbI_3$ perovskite sample. Such images were acquired at the different locations on the undoped samples, and I-V, dI/dV, and d^2I/dV^2 tunneling spectroscopy data were simultaneously recorded on 8 x 8 grid points at each surface area. Here, the distances between the grid points are 2 nm x 2 nm. The tunneling spectroscopy data were recorded under 400 nm illumination, and the undoped samples usually provide a bandgap and exhibit a semiconducting behavior. An example spectroscopy data sequence measured at this sample location is provided below.

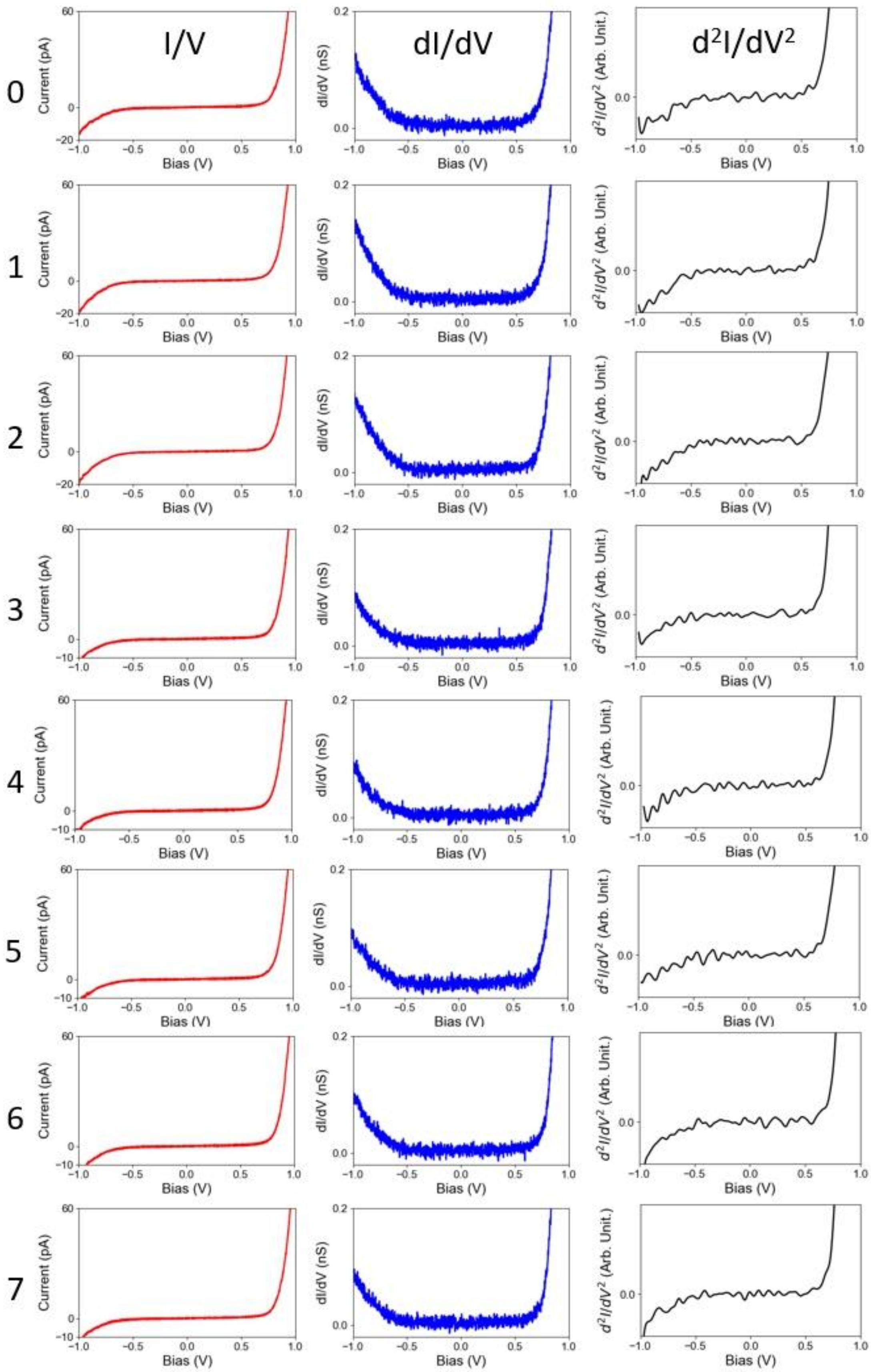

I/V
dI/dV
d²I/dV²
0
1
2
3
4
5
6
7
Current (pA)
dI/dV (nS)
d²I/dV² (Arb. Unit.)
Bias (V)

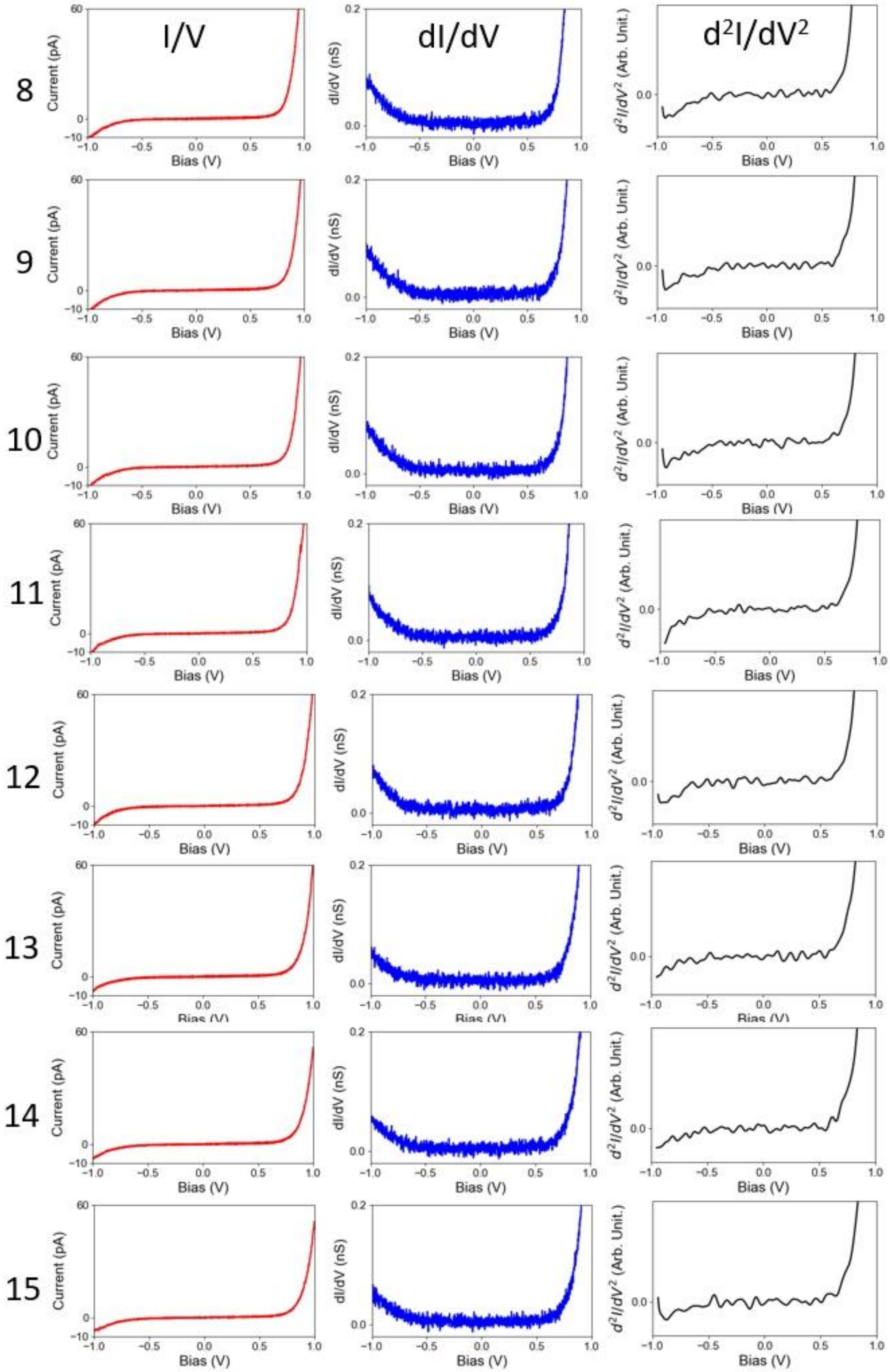
I/V
dI/dV
d2I/dV2
8
9
10
11
12
13
14
15
Current (pA)
dI/dV (nS)
d2I/dV2 (Arb. Unit.)
Bias (V)

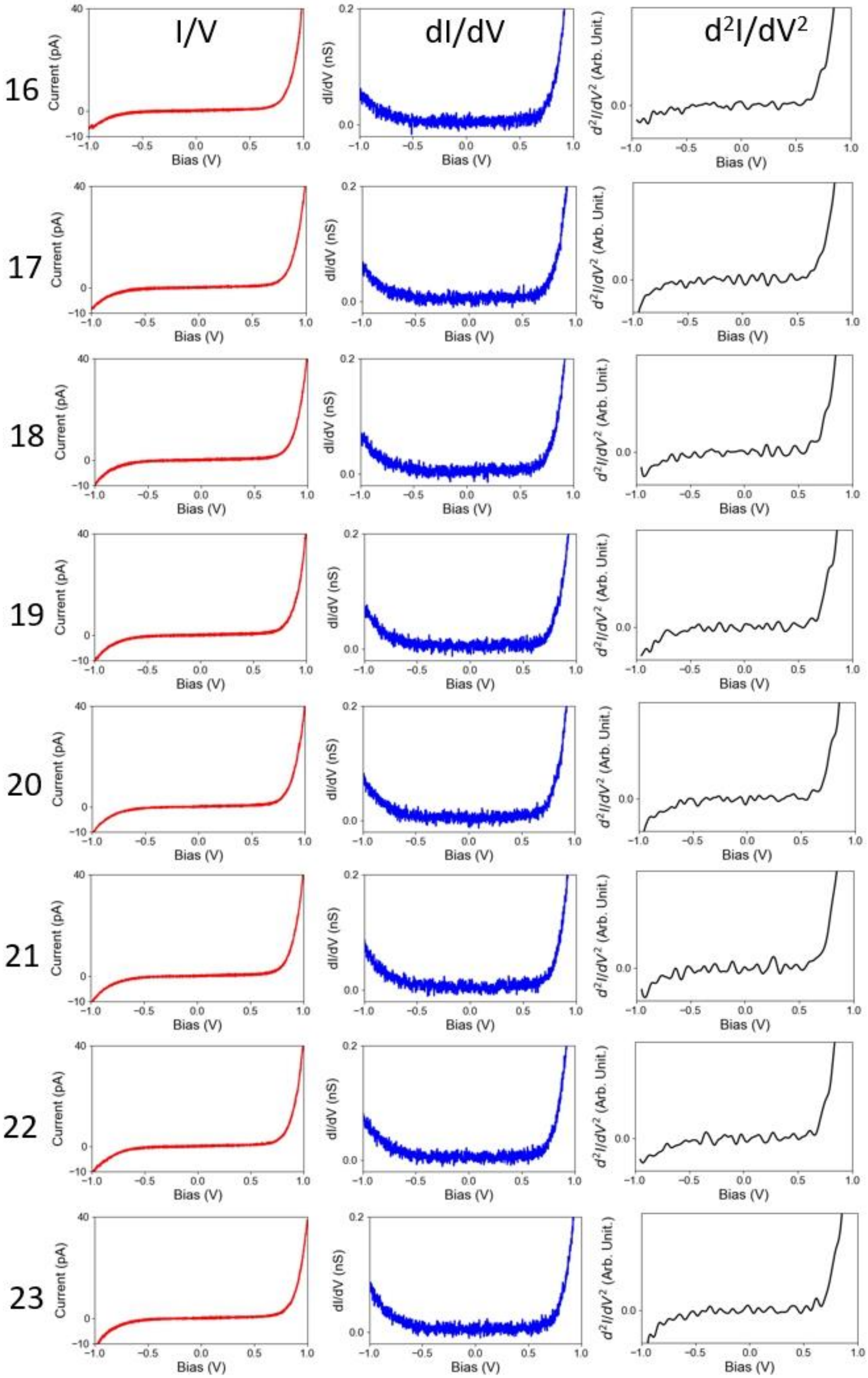

I/V
dI/dV
d²I/dV²
16
17
18
19
20
21
22
23
Current (pA)
dI/dV (nS)
d²I/dV² (Arb. Unit.)
Bias (V)

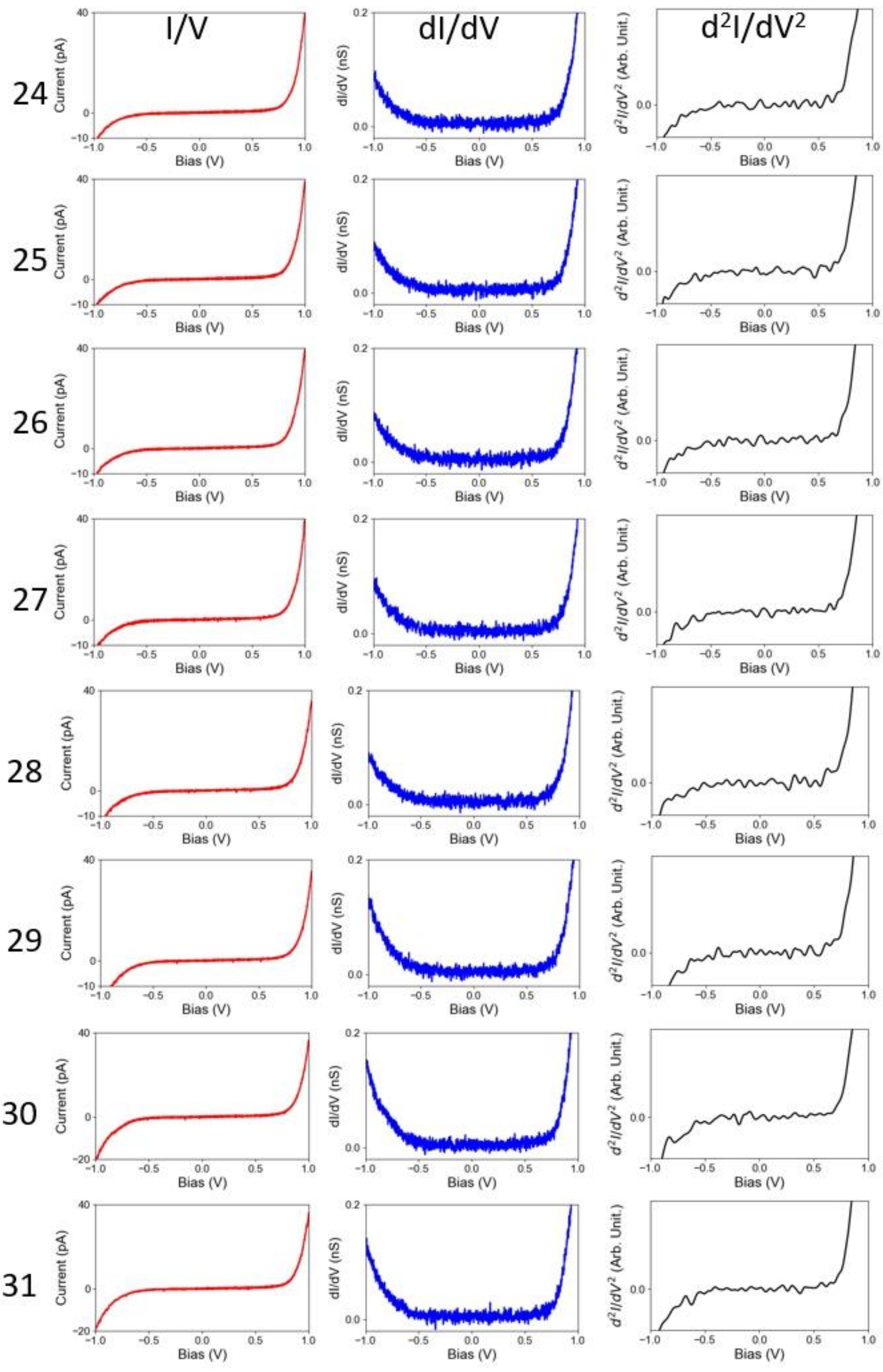
I/V
dI/dV
d²I/dV²
24
25
26
27
28
29
30
31
Current (pA)
dI/dV (nS)
d²I/dV² (Arb. Unit.)
Bias (V)

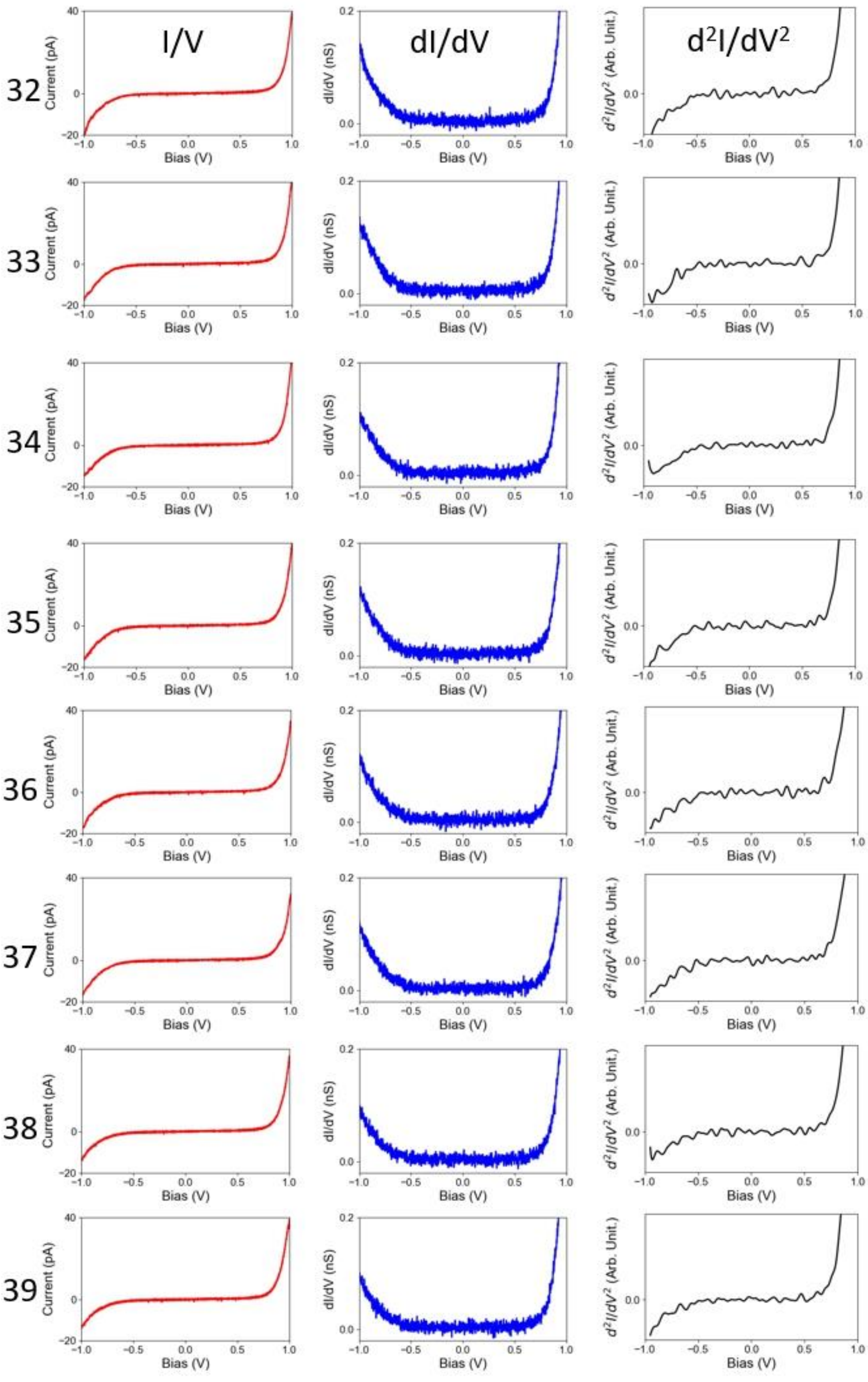
I/V
dI/dV
d²I/dV²
32
33
34
35
36
37
38
39
Current (pA)
dI/dV (nS)
d²I/dV² (Arb. Unit.)
Bias (V)

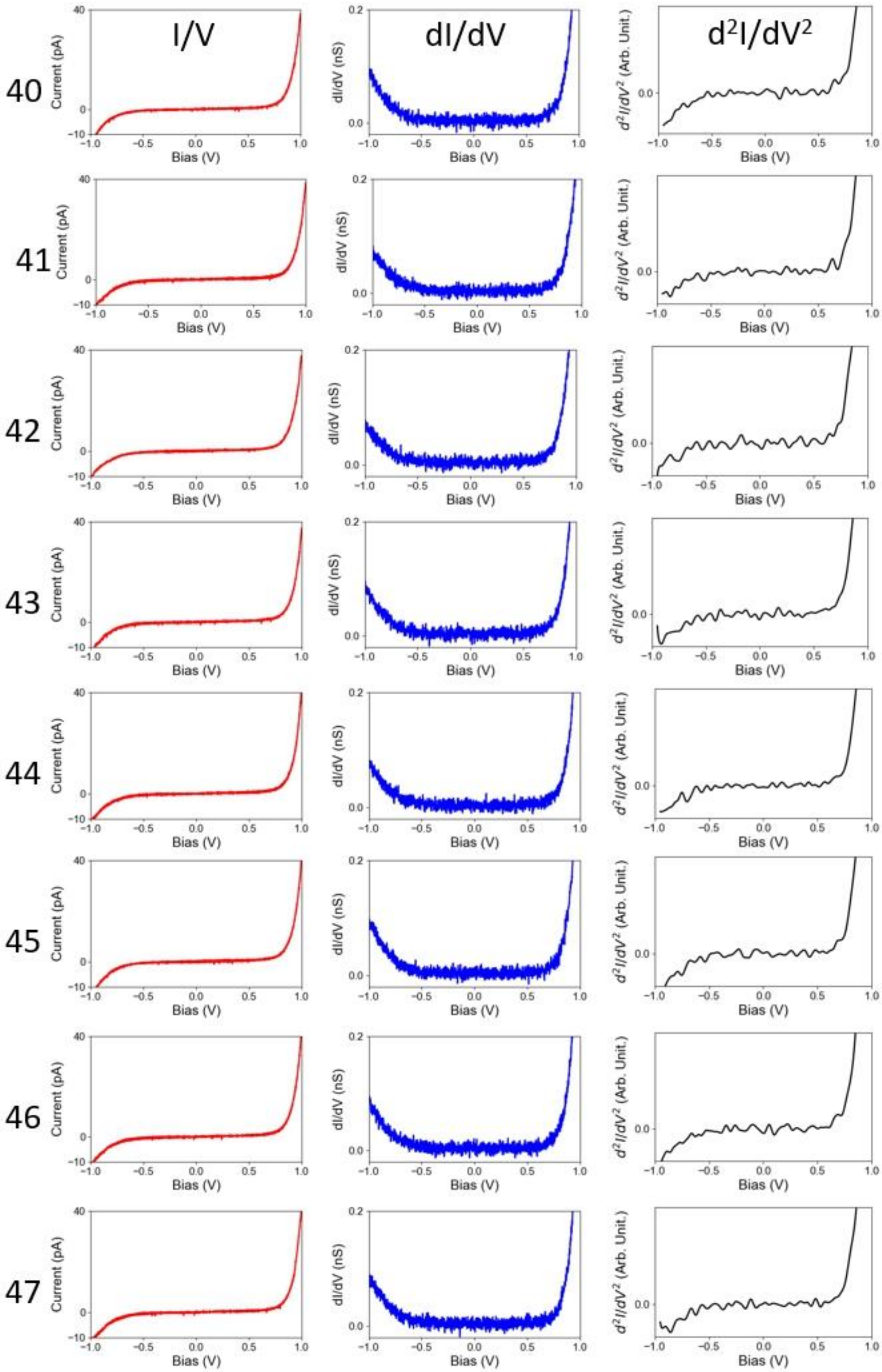
I/V
dI/dV
d2I/dV2
40
41
42
43
44
45
46
47
Current (pA)
dI/dV (nS)
d2I/dV2 (Arb. Unit.)
Bias (V)

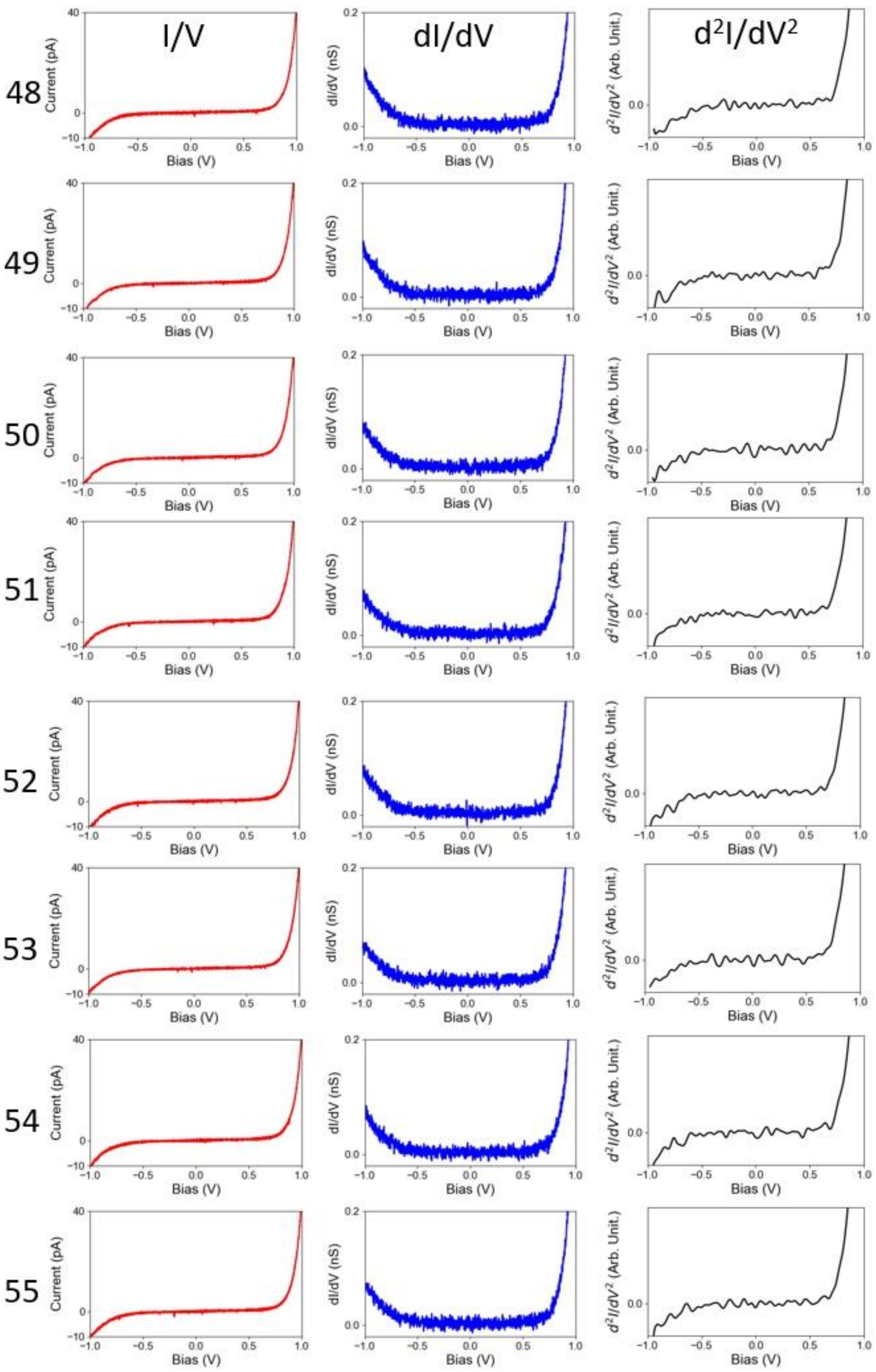
I/V
dI/dV
d2I/dV2
48
49
50
51
52
53
54
55
Current (pA)
dI/dV (nS)
d2I/dV2 (Arb. Unit.)
Bias (V)

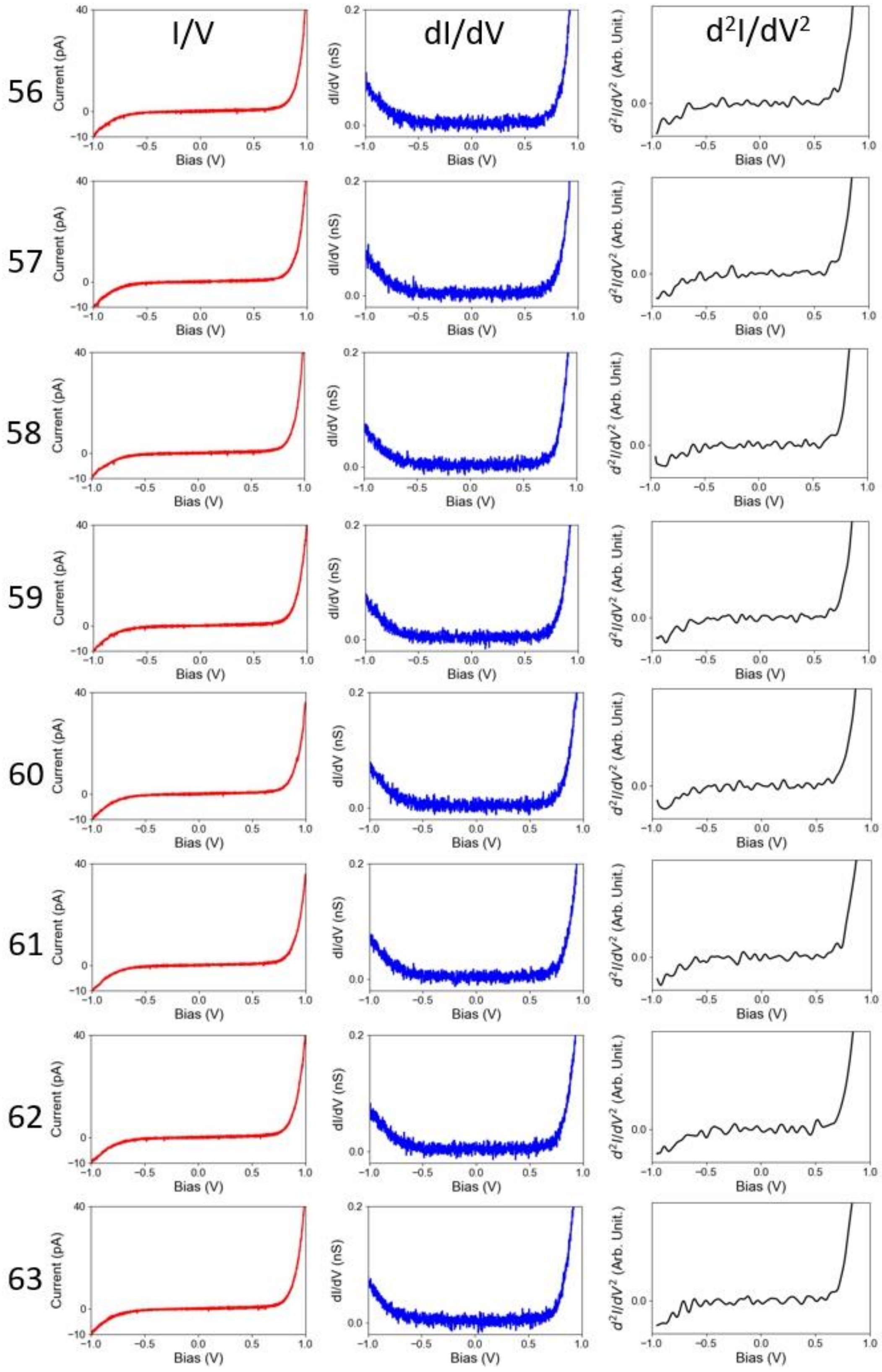

Supplementary Fig. 28. I-V (left), dI/dV (middle), and d^2I/dV^2 (right) curves of pristine $MAPbI_3$ perovskite with spatially periodic sampling.

Supplementary Table 1. ICP-MS analysis results of concentration of Pb and Nd elements in perovskite film.

Concentration of HNO_3-digested perovskite solution in ultrapure water	Concentration of Nd (μg/L)	Concentration of Pb (μg/L)
2000 ppm	3.5	230
1000 ppm	1.8	110
100 ppm	0.21	13

The result suggests that the molar ratio of Nd: Pb in 2%Nd:$MAPbI_3$ film is approximately 2.2:100.

Supplementary Table 2. Lattice parameter comparison between $MAPbI_3$, 2% Nd:$MAPbI_3$ through Rietveld refinement based on the standard powder diffraction file (PDF) of $MAPbI_3$ (COD: 7218931).

Composition	*a* (Å)	*c* (Å)	α (degree)	β (degree)	γ (degree)	Volume ($Å^3$)
$MAPbI_3$	8.9060	12.5890	90º	90º	90º	998.5643
2% Nd:$MAPbI_3$	8.9109	12.5960	90º	90º	90º	1000.1746
PDF 7218931	8.896	12.637	90	90	90	1000.1

Supplementary Table 3. X-ray Powder Diffraction peak search report and crystallinity calculated by Scherer equation.

a, **Pristine $MAPbI_3$ sample:** average crystallite is 71.25 (nm)

2-Theta (degree)	d-spacing (nm)	Height (a.u.)	FWHM (degree)	Crystallite (nm)
14.183	6.2396	6099	0.111	71.97
28.413	3.1387	814	0.093	87.15
31.983	2.7960	375	0.096	85.14
34.930	2.5665	370	0.258	31.93
40.520	2.2244	626	0.141	59.46
43.106	2.0968	613	0.092	91.82

b, **2%Nd:$MAPbI_3$ sample:** average crystallite is 77.87（nm)

2-Theta (degree)	d-spacing (nm)	Height (a.u.)	FWHM (degree)	Crystallite (nm)
14.019	6.3121	6725	0.099	79.96
28.270	3.1542	4448	0.094	86.19
31.830	2.8091	298	0.112	72.95
34.798	2.5760	694	0.151	54.53
40.499	2.2255	595	0.105	79.76
43.080	2.0980	705	0.090	93.85
